# Immunological shifts during early-stage Parkinson’s disease identified with DNA methylation data on longitudinally collected blood samples

**DOI:** 10.1038/s41531-023-00626-6

**Published:** 2024-01-11

**Authors:** Steven C. Pike, Matthew Havrda, Francesca Gilli, Ze Zhang, Lucas A. Salas

**Affiliations:** 1 Integrative Neuroscience at Dartmouth, Guarini School of Graduate and Advanced Studies at Dartmouth College, Hanover, NH USA; 2 Department of Epidemiology, Geisel School of Medicine at Dartmouth College, Lebanon, NH USA; 3 Department of Neurology, Dartmouth Hitchcock Medical Center, Lebanon, NH USA; 4 Department of Molecular and Systems Biology, Geisel School of Medicine at Dartmouth College, Hanover, NH USA

**Keywords:** Parkinson's disease, Genetic databases, Epidemiology

## Abstract

Parkinson’s disease (PD) is the second most common neurodegenerative disease in the United States. Decades before motor symptoms manifest, non-motor symptoms such as hyposmia and rapid eye movement (REM) sleep behavior disorder are highly predictive of PD. Previous immune profiling studies have identified alterations to the proportions of immune cells in the blood of clinically defined PD patients. However, it remains unclear if these phenotypes manifest before the clinical diagnosis of PD. We utilized longitudinal DNA methylation (DNAm) microarray data from the Parkinson’s Progression Marker’s Initiative (PPMI) to perform immune profiling in clinically defined PD and prodromal PD patients (Prod). We identified previously reported changes in neutrophil, monocyte, and T cell numbers in PD patients. Additionally, we noted previously unrecognized decreases in the naive B cell compartment in the defined PD and Prod patient group. Over time, we observed the proportion of innate immune cells in PD blood increased, but the proportion of adaptive immune cells decreased. We identified decreases in T and B cell subsets associated with REM sleep disturbances and early cognitive decline. Lastly, we identified increases in B memory cells associated with both genetic (*LRRK2* genotype) and infectious (cytomegalovirus seropositivity) risk factors of PD. Our analysis shows that the peripheral immune system is dynamic as the disease progresses. The study provides a platform to understand how and when peripheral immune alterations occur in PD and whether intervention at particular stages may be therapeutically advantageous.

## Introduction

Parkinson’s disease (PD) is an age-related neurodegenerative disorder with multisystemic manifestations characterized histopathologically by the loss of dopaminergic (DA) neurons in the substantia nigra pars compacta^1^. PD-related proteinopathy is characterized by the identification of aggregates of alpha-synuclein protein and alpha-synuclein immunoreactive Lewy Bodies in brain regions near and distal to the sites of neurodegeneration^2^. The diagnostic criteria for PD rely on the clinical determination of bradykinesia, in conjunction with rest tremor or rigidity, while ruling out other potential causes^3,4^. Before the onset of motor symptoms and years before clinical diagnosis, non-motor symptoms such as REM sleep behavior disorder (RBD) and hyposmia are common^5–7^. Patients who experience these symptoms but do not exhibit clear motor deficits are considered to be in the prodromal phase of PD^7^.

Inflammation is an easily identified histopathological component of PD^8^. Current data support the role of inflammation in the pathogenesis of PD citing microglial activation^9^, the identification of genetic variation in genes such as HLA-DR^10,11^, and evidence of immune infiltration in the brain parenchyma^12–15^. Interactions between the peripheral immune system and PD are poorly understood but analysis of post-mortem tissue, cerebrospinal fluid (CSF), and blood have provided evidence of complex interactions between peripheral, local, innate, and adaptive immune responses during the neurodegenerative process^16^.

The innate immune system provides an early-stage line of defense against infectious agents or damage to other cells in the body. Broadly, these cells recognize damaged neurons in the PD brain, clear debris, and relay the signal to the periphery^17^. Microglia are the central nervous system (CNS) resident innate immune cells, while myeloid (neutrophils, basophils, eosinophils, and monocytes) and natural killer (NK) cells are those in the blood. Microglia detect PD-related damage-associated molecular patterns (DAMPs) such as alpha-synuclein proteinopathy or neuronal damage and relay signals to peripheral immune cells^17^. However, the role of peripheral immune cells in initiating or propagating inflammatory damage in the CNS of PD patients is not entirely known. Neutrophils can increase the permeability of the blood-brain barrier (BBB), giving all immune cells increased access to the CNS^18^. Evidence for neutrophil activity in the PD brain is based on the observed expression of myeloperoxidase^19^. Basophils and eosinophils are specialized in responses to allergens and parasitic infections; some associations have been reported between asthma or allergies to plants, pollen, or antibiotics and an increased risk of PD^20,21^. Monocytes are phagocytes that can clear debris and recruit immune cells. In PD, monocytopenia, increased phagocytic behavior, and decreased viability and cytokine production upon ex vivo stimulation have been observed^22–24^. Lastly, NK cells recognize non-specific biomarkers and have been thought to aid in the clearance of alpha-synuclein and communication with dying neurons^25^. Others have noted that the activity and numbers of NK cells increase as PD progresses^22,26–29^.

The adaptive immune system provides a secondary line of defense against antigens and produces a memory response facilitated by T and B cells. Recent work has suggested both pathogenic and neuroprotective roles of the adaptive immune cells in various neurodegenerative contexts^30^. T cells have been identified within the substantia nigra of PD patients^31–33^. Additionally, PD patients possess CD4^+^ T cells that are primed to a Th1 proinflammatory phenotype and react to epitopes of alpha-synuclein^34–36^. B cells are the antibody-producing cells of the body. In PD, antibodies specific to alpha-synuclein have been identified in patient serum and CSF^37,38^. Whether an inflammatory immune response is a cause or a consequence of tissue damage in neurodegenerative diseases like PD is still controversial. However, it is agreed that the CNS has a direct line of communication with the peripheral immune system, and intercepting these signals may allow us to monitor CNS disease states from the blood^39^.

Immune profiling on an epidemiological scale is challenging due to the lack of fresh samples that can be processed using flow/mass cytometry. Epigenomics technology has opened the potential to scale this type of data collection without sacrificing resolution. DNA methylation (DNAm) is an epigenetic marker placed on cytosine bases and is used to regulate gene expression and cell differentiation^40^; cell-type specific genes can be expressed and are hypomethylated, while other genes are repressed with hypermethylation of regulatory gene elements^41^. These durable, intrinsic lineage markers allow researchers to use DNAm data to estimate the composition of a specimen by using cell-type specific DNAm profiles^42–45^. This approach, known as cellular deconvolution, can be used to assess compositional differences as a potential element of the disease processes and allow for control of confounding by cellular heterogeneity across samples in epigenome-wide association studies (EWAS)^46^.

Several studies have already performed immune profiling of PD blood samples using conventional methods such as flow cytometry and DNAm data^23,47–54^. However, our current knowledge of the peripheral immune dynamics in PD is limited. Firstly, many of these studies investigated broad proportions of immune cells where details about subsets of B cells, T cells, and granulocytes may have been lost due to technical limitations^23,52^. Similarly, previous DNA-based assessments have utilized earlier versions of deconvolution platforms that also do not investigate some of these cell subtypes^49^. Secondly, many of these studies have assessed immune dysregulation in clinically defined PD cases; nonetheless, we know that CNS damage may begin decades before motor symptom onset^55^. There is a significant gap in our understanding of when precisely the peripheral immune perturbations manifest concerning the onset of neurological damage rather than a clinical diagnosis of PD.

To fill this gap, we utilized the data available from the Parkinson’s Progression Markers Initiative (PPMI) to study the early immunological perturbations during PD. The PPMI is a large PD case-control study implemented to identify PD biomarkers^56,57^. The PPMI collected a battery of longitudinal data, including whole blood DNA data, from PD patients, healthy participants, and a group of prodromal PD patients^58^. Our goal is to perform an in-depth analysis of cellular composition using the PPMI data with a deconvolution library developed and validated by our laboratory that extends to 12 cell types^42^. We can assess how early the immunological perturbations manifest in the PD progression timeline by analyzing the prodromal PD patients in parallel to the clinically defined PD patients. We hypothesize that immunological dysregulation will manifest alongside the earliest clinically observable events, and thus, we will see peripheral immunological perturbations in prodromal PD patients.

## Results

### Patient data

Participants selected for this analysis were assigned to one of three patient groups (Table 1). The healthy control group (HC) were neurologically healthy participants of similar demographics to the case groups. The Parkinson’s disease group (PD) consisted of participants diagnosed with PD less than two years before the screening visit and were naive to PD-specific medications. The prodromal PD group (Prod) consisted of participants who exhibited symptoms indicative of early stages of PD or tested positive for PD genetic risks alleles (Table 2). After enrollment, blood DNAm and clinical data were collected annually.

**Table 1:** Baseline characteristics of the subset of PPMI participants included in this analysis.

	Healthy Controls (HC)	Prodromal (Prod)	Parkinson’s Disease (PD)	p-value	p-value symbol	Statistical Test
*n*	83	175	298			
**Biographical**						
Female (%)	28 (33.7)	84 (48.0)	115 (38.6)	0.048	*	a
Pre-menopause (% of Female)	1 (0.4)	2 (2.4)	6 (5.2)	0.73		a
Age (mean (SD))	61.18 (10.48)	63.43 (7.26)	62.14 (9.47)	0.133		c
Self-reported race (%)				0.046	*	a
Race other than white	8 (9.6)	5 (2.9)	10 (3.4)			
White	75 (90.4)	169 (96.6)	288 (96.6)			
Not Specified	0 (0.0)	1 (0.6)	0 (0.0)			
Hispanic/Latino (%)				<0.001	***	a
No	81 (97.6)	146 (83.4)	278 (93.3)			
Yes	1 (1.2)	29 (16.6)	20 (6.7)			
Not Specified	1 (1.2)	0 (0.0)	0 (0.0)			
**DaT Scan Imaging**						
DaT Scan Positive (%)	1 (1.2)	—	289 (99.7)			
Putamen						
Right SBR (median [IQR])	2.07 [1.73, 2.47]	—	0.75 [0.57, 1.00]	<0.001	***	b
Left SBR (median [IQR])	2.00 [1.73, 2.50]	—	0.73 [0.57, 0.95]	<0.001	***	b
AsI (median [IQR])	5.11 [2.60, 8.28]	—	16.50 [7.45, 25.44]	<0.001	***	b
Caudate						
Right SBR (median [IQR])	2.79 [2.50, 3.28]	—	1.93 [1.52, 2.33]	<0.001	***	b
Left SBR (median [IQR])	2.82 [2.51, 3.28]	—	1.99 [1.56, 2.30]	<0.001	***	b
AsI (median [IQR])	3.46 [1.92, 5.76]	—	8.80 [4.71, 13.35]	<0.001	***	b
**Clinical Survey Scores**						
MDS-UPDRS Part 1 (median [IQR])	2.00 [1.00, 4.00]	3.00 [1.00, 6.00]	4.00 [2.00, 7.00]	<0.001	***	b
MDS-UPDRS Part 2 (median [IQR])	0.00 [0.00, 0.50]	0.00 [0.00, 2.00]	5.00 [3.00, 8.00]	<0.001	***	b
ESS (median [IQR])	4.00 [3.00, 7.00]	5.00 [3.00, 8.00]	6.00 [3.00, 8.00]	0.056		b
MoCA (median [IQR])	28.00 [27.00, 29.00]	27.00 [25.00, 29.00]	27.00 [26.00, 29.00]	<0.001	***	b
GDS (median [IQR])	1.00 [1.00, 2.00]	1.00 [1.00, 2.00]	2.00 [1.00, 3.00]	0.131		b
STAI (median [IQR])	53.00 [45.25, 63.00]	57.00 [48.00, 70.75]	64.00 [52.00, 78.00]	<0.001	***	b
REM (median [IQR])	2.00 [1.00, 4.00]	4.00 [2.00, 6.00]	4.00 [2.00, 6.00]	<0.001	***	b
**Samples per Study Visit**						
BL (baseline)	83	175	298			
V03 (9 months)	1	0	4			
V04 (12 months)	81	172	276			
V05 (18 months)	1	5	12			
V06 (24 months)	79	170	278			
V07 (30 months)	2	31	19			
V08 (36 months)	77	126	271			
V09 (42 months)	4	6	13			
Samples per patient (median [IQR])	4 [4, 4]	4 [4, 4]	4 [4, 4]			

Statistical tests *a* = chi-squared, *b* = Kruskal–Wallis Rank Sum Test, *c* = One-way ANOVA.

*SD* standard deviation, *IQR* inter-quartile range, *SBR* signal binding ratio, *AsI* asymmetry index, *MDS-UPDRS1* Movement Disorder Society Unified Parkinson’s Disease Rating Scale Part 1 (total score), *MDS-UPDRS2* Movement Disorder Society Unified Parkinson’s Disease Rating Scale Part 2 (total score), *ESS* Epworth Sleepiness Scale (total score), *MoCA* Montreal Cognitive Assessment (total score), *STAI* State-Trait Anxiety Inventory (total score), *REM* REM Sleep Behavior Questionnaire (total score), *GDS* Geriatric Depression Scale (total score), *VXX* PPMI visit number XX.

**Table 2:** Prod group inclusion criteria.

	Healthy Controls (HC)	Prodromal (Prod)	Parkinson’s Disease (PD)
*n*	83	175	298
Prodromal Inclusion Criterion			
Any 1st Degree Family with PD (%)	6 (7.2)	7 (4.0)	52 (17.4)
Hyposmia (%)			
Not Available	—	2 (1.1)	—
No	—	122 (69.7)	—
Yes	—	51 (29.1)	—
REM Sleep Behavior Disorder (%)	—	28 (16.0)	—
Genotype			
LRRK2 Risk Allele Carriers (%)	0 (0.0)	127 (72.6)	84 (28.2)
GBA Risk Allele Carriers (%)	0 (0.0)	11 (6.3)	12 (4.0)
SNCA Risk Allele Carriers (%)	0 (0.0)	0 (0.0)	0 (0.0)
Converted to PD (%)			
Not Available	—	2 (1.1)	—
No	—	153 (87.4)	—
Yes	—	20 (11.4)	—

Participants were included in the Prod group based on the presence of symptoms or high-risk genetic alleles.

**Fig. 1:**
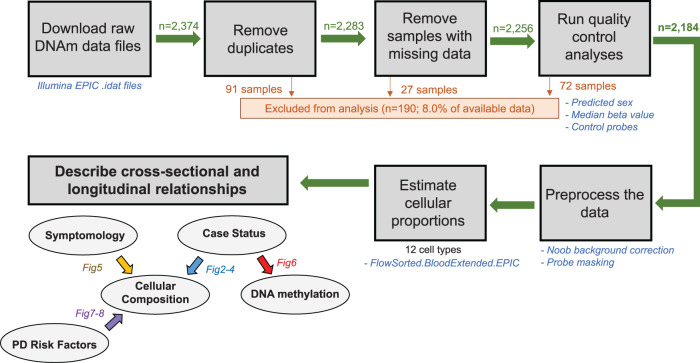
Data analysis and sample exclusion pipeline. All available DNAm data files were downloaded from PPMI, but after our quality control analyses, we included 2184 samples in the study. Using the DNAm data, we estimated the proportions of immune cells in each blood sample and performed different epidemiological analyses, denoted by the figure number in the abbreviated causal diagram.

Raw bulk DNAm data were downloaded from the PPMI database and analyzed (Fig. 1). After the quality control assessment, we included 2184 samples in our study (Supplementary Table 1). The *FlowSorted.BloodExtended.EPIC* package was used to deconvolute each blood sample, i.e., calculate the proportions of immune cells^42^: this includes basophils (Bas), naive and memory B cells (Bnv, Bmem), naive and memory CD4^+^T cells (CD4nv, CD4mem), naive and memory CD8^+^T cells (CD8nv, CD8mem), eosinophils (Eos), monocytes (Mono), neutrophils (Neu), natural killer cells (NK), and T regulatory cells (Treg). These values were then adjusted to account for each cell type’s limit of detection (LoD, see Methods). Because 98.5% of samples were below the LoD for Tregs, these cells were excluded from all subsequent analyses (Supplementary Fig. 1).

We noticed two batch effects present in the dataset (Supplementary Fig. 2). The first was associated with the DNAm data processing batch; the second was associated with the run date of the array. Each of these variables was transformed into binary variables and they were included as covariates in all subsequent analyses. Sensitivity analyses confirmed our findings were valid in a subset of data that did not include these batch effects (data not shown).

### Comparing the cellular proportions of PD and Prod groups to HC

**Fig. 2:**
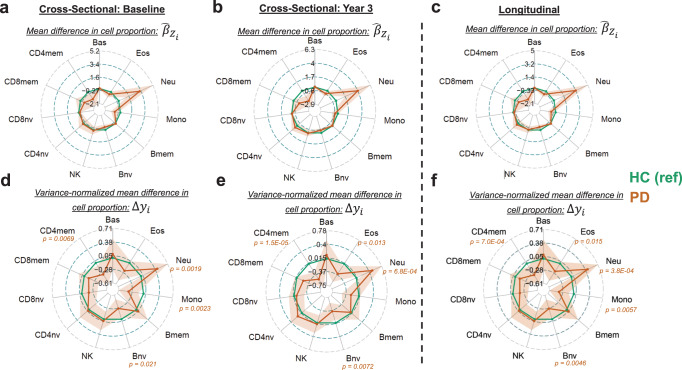
Comparing the DNAm derived immune cell profile of PD patients to age and sex-matched healthy controls. Plotted is the effect size and 95% confidence interval for having PD compared to HC in our regression models for each cell type. **a**–**c** plot the effect size as derived from the models, whereas (**d**–**f**) plot the variance normalize mean difference. **a**, **b**, **d**, **e** were derived using linear models, whereas (**c**, **f**) were derived using mixed effect regression models. Models were adjusted for age, sex, and batch variables. Only *p* values <0.05 are denoted (regression model *t* test; df = 375 for (**a**, **d**); df = 342 for (**b**, **e**); df = 385.3 for (**c**, **f**)). HC healthy control group, PD Parkinson’s disease group, Bas basophils, Eos eosinophils, Neu neutrophils, Bnv B-cells naïve, Bmem B-cells memory, CD4nv helper CD4^+^ T-cells naïve, CD4mem helper CD4^+^ T-cells memory, Treg CD4^+^ T regulatory cells, CD8nv cytotoxic CD8^+^ T-cells naïve, CD8mem cytotoxic CD8^+^ T-cells memory, Mono monocytes, NK natural killer cells, df degrees of freedom.

We performed regression analyses to estimate the effect of PD on the proportion of each immune cell. Our regression analyses were adjusted for the patient’s age at their baseline visit, sex, and batch effects. We conducted two parallel analyses using cross-sectional and longitudinal approaches. This dual strategy captures inter-timepoint dynamics across linear cross-sectional models at each study time point. We then utilized a longitudinal model to have a well-powered estimate for the overall effect of having PD on the immune proportion. Radar plots show the average difference in cell proportion between the PD group and the HC group at the BL sample (Fig. 2a) and at the final major study timepoint (Fig. 2b). We performed a single-sample t-test for each effect size to test if the estimate was non-zero and generate a *p*-value. We used a linear mixed effects model for the longitudinal approach, including a random effect for each patient to account for the repeated measures and time as an additional variable (Fig. 2c).

We anticipated that comparing the linear effect sizes from these models would visually inflate the changes to abundant cell types such as Neu. To calibrate the effect sizes, we divided each by the standard deviation of the cell proportion in the HC group (Fig. 2d–f). These values represent the mean difference in cell proportion in the units of the number of standard deviations away from the healthy population (Eq. II, see Methods). This data representation allows us to visualize small yet significant changes in lowly abundant cell types, such as Bnv cells.

Consistent across both cross-sectional and longitudinal methods, we found that the PD group had lower proportions of CD4mem and Bnv and higher proportions of Neu. We also identified a decrease in Eos (${\hat{\beta }}_{Z}$ = -0.48, *p* = 0.015) and Mono (${\hat{\beta }}_{Z}$ = -0.61, *p* = 0.0057) proportions in the longitudinal model. At the baseline visit, we found a decrease in Mono proportions (${\hat{\beta }}_{Z}$ = -0.79, *p* = 0.0023); however, it was no longer significant at the Year 3 time point (${\hat{\beta }}_{Z}$ = -0.45, *p* = 0.082).

**Fig. 3:**
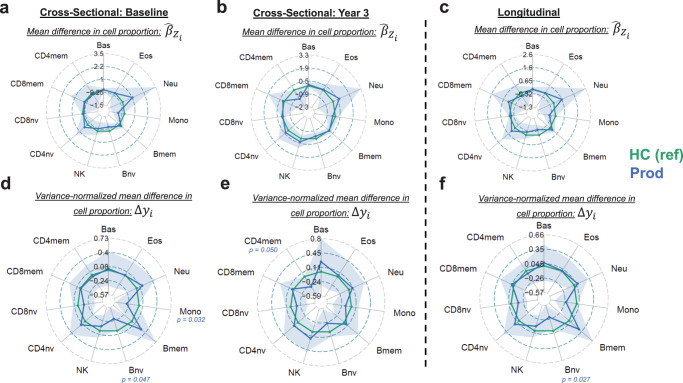
Comparing the DNAm derived immune cell profile of Prod patients to age and sex matched healthy controls. Plotted is the effect size and 95% confidence interval for having Prod compared to HC in our regression models for each cell type. **a**–**c** plot the effect size as derived from the models, (**d**–**f**) plot the variance normalized mean difference. **a**, **b**, **d**, **e** were derived using linear models, whereas (**c**, **f**) were derived using mixed effect regression models. Models were adjusted for age, sex, and batch variables. Only *p* values <0.05 are denoted (regression model *t* test; df = 252 for (**a**, **d**); df = 197 for (**b**, **e**); df = 262.6 for (**c**, **f**)). HC healthy control group, Prod prodromal Parkinson’s disease group, Bas basophils, Eos eosinophils, Neu neutrophils, Bnv B-cells naïve, Bmem B-cells memory, CD4nv helper CD4^+^ T-cells naïve, CD4mem helper CD4^+^ T-cells memory, Treg CD4^+^ T regulatory cells, CD8nv cytotoxic CD8^+^ T-cells naïve, CD8mem cytotoxic CD8^+^ T-cells memory, Mono monocytes, NK natural killer cells, df degrees of freedom.

We employed the same dual approaches to compare the cellular composition of the Prod group to HC (Fig. 3). We first looked at yearly time points in the study (Fig. 3a, b) in addition to a longitudinal model (Fig. 3c). At the baseline time point, we observed a decrease in Bnv (${\hat{\beta }}_{Z}$ = -0.42, *p* = 0.047) and Mono (${\hat{\beta }}_{Z}$ = -0.62, *p* = 0.031) (Fig. 3d). Although not statistically significant, we observed an increase in Bmem cells (${\hat{\beta }}_{Z}$ = 0.19, *p* = 0.21). At the Year 3 time point, we found a decrease in CD4mem cells (${\hat{\beta }}_{Z}$ = -1.2, *p* = 0.050) (Fig. 3e). Looking at the longitudinal model, we observe an overall reduction in Bnv cells (${\hat{\beta }}_{Z}$ = -0.42, *p* = 0.027) (Fig. 3f). We also identified strong trends in Bmem (${\hat{\beta }}_{Z}$ = 0.21, *p* = 0.15) and Mono (${\hat{\beta }}_{Z}$ = -0.47, *p* = 0.059) in the longitudinal model.

We directly compared the PD and Prod groups to each other to identify changes in cell composition that may differ between pre-motor deficit PD and clinically defined PD (Supplementary Fig. 3). We identified that the increased levels of Neu is specific to the clinically defined PD patients (${\hat{\beta }}_{Z}$ = 2.2, *p* = 0.0032 in the longitudinal model). We also observe marginal decreases in CD4T cell populations in the PD group compared to the Prod group.

**Fig. 4:**
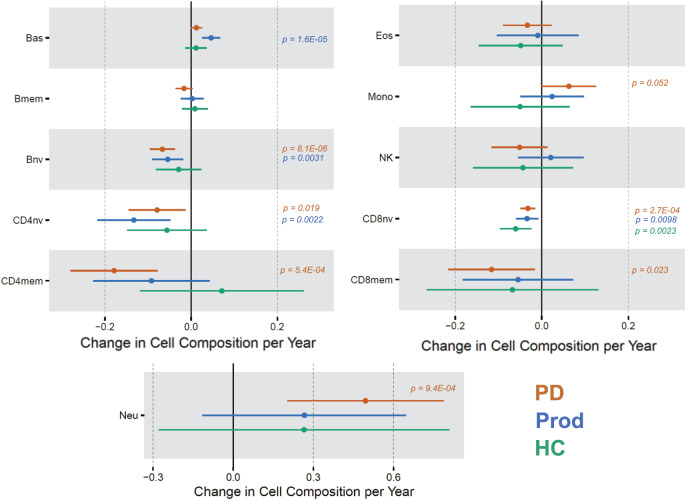
Rate of change of change in DNAm derived cell composition in each cohort. Plotted is the effect size and 95% confidence interval for the effect of time on the level of each cell type in the mixed effect longitudinal models. We stratified the data and ran a model for each patient group to identify a potential interaction. Each model was adjusted for age, sex, and batch variables. Only *p* values <0.05 are denoted (regression model *t* test; df = 243.7 for HC; df = 867.7 for PD; df = 507.0 for Prod). HC healthy control group, PD Parkinson’s disease group, Prod prodromal Parkinson’s disease group, Bas basophils, Eos eosinophils, Neu neutrophils, Bnv B-cells naïve, Bmem B-cells memory, CD4nv helper CD4^+^ T-cells naïve, CD4mem helper CD4^+^ T-cells memory, Treg CD4^+^ T regulatory cells, CD8nv cytotoxic CD8^+^ T-cells naïve, CD8mem cytotoxic CD8^+^ T-cells memory, Mono monocytes, NK natural killer cells, df degrees of freedom.

Because we obtained different results across cross-sectional time points, we assessed how the cell proportions changed over time in each group and determined if they changed at different rates using stratified longitudinal models (Fig. 4). Interestingly, we observed a decrease in CD8nv cells over time in all groups (HC ${\hat{\beta }}_{{time}}$ = -0.060, *p* = 0.0013; Prod ${\hat{\beta }}_{{time}}$ = -0.034, *p* = 0.0098; PD ${\hat{\beta }}_{{time}}$ = -0.032, *p* = 2.7E-4). For the Prod group, we also observed an increase in Bas (${\hat{\beta }}_{{time}}\,$= 0.046, *p* = 1.6E-5), and a decrease in Bnv (${\hat{\beta }}_{{time}}\,$= -0.054, *p* = 0.0031) and CD4nv cells (${\hat{\beta }}_{{time}}$ = -0.13, *p* = 0.0022). For the PD group, we observed a decrease in Bnv (${\hat{\beta }}_{{time}}$ = -0.067, *p* = 8.1E-6), CD4nv (${\hat{\beta }}_{{time}}\,$= -0.079, *p* = 0.019), CD4mem (${\hat{\beta }}_{{time}}$ = -0.18, *p* = 5.4E-4), and CD8mem (${\hat{\beta }}_{{time}}$ = -0.12, *p* = 0.023) over time, while we saw an increase in Mono (${\hat{\beta }}_{{time}}\,$= 0.063, *p* = 0.052) and Neu (${\hat{\beta }}_{{time}}$ = 0.49, *p* = 9.4E-4).

To formally test if the rate of change in the PD or Prod group is unique from the HC group, we performed likelihood ratio tests between models that did and did not include an interaction term for case status and time. A significant result implies that allowing the model to fit unique trends over time for each group (interaction model) better represents the data compared to a model that tries to fit one trend for the entire dataset (additive model). Comparing PD to HC, the likelihood ratio test was significant for Bas (*p* = 0.0097), Bmem (*p* = 0.036), and CD8nv (*p* = 0.046). Comparing Prod to HC, the likelihood ratio test was significant for Bmem (*p* = 0.016), Bnv (*p* = 0.035), and CD8nv (*p* = 0.036).

**Fig. 5:**
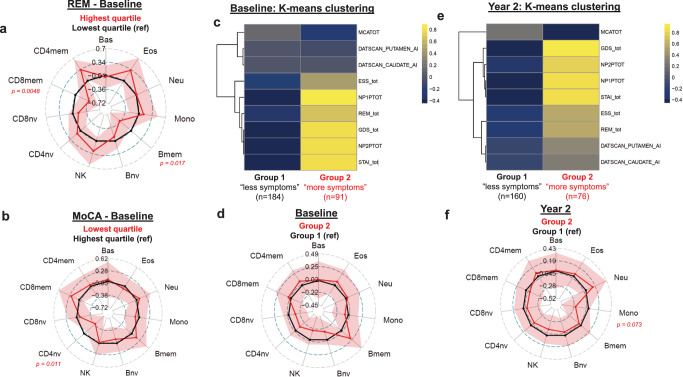
DNAm derived immune cell proportions correlate with PD clinical scores. All radar plots represent variance normalized mean difference in cell proportion with 95% confidence intervals. **a**, **b** show relationships between specific clinical variables and the proportions of each cell type in which PD patients in the most symptomatic quartile are compared to PD patients in the least symptomatic quartile. **c**, **e** are heatmaps of the mean value of each clinical score in each cluster identified by the clustering approach. All scores were mean scaled and centered before clustering, color scale shows scaled *Z*-score. **d**, **f** show the comparison of group 2 to group 1 immune cell profiles. Models were adjusted for age, sex and batch variables. Only *p* values <0.05 are denoted (regression model *t* test; df = 271 for (**a**); df = 268 for (**b**); df = 269 for (**d**); df = 230 for (**f**)). Bas basophils, Eos eosinophils, Neu neutrophils, Bnv B-cells naïve, Bmem B-cells memory, CD4nv helper CD4^+^ T-cells naïve, CD4mem helper CD4^+^ T-cells memory, Treg CD4^+^ T regulatory cells, CD8nv cytotoxic CD8^+^ T-cells naïve, CD8mem cytotoxic CD8^+^ T-cells memory, Mono monocytes, NK natural killer cells, AI asymmetry index, NP1PTOT Movement Disorder Society Unified Parkinson’s Disease Rating Scale Part 1 (total score), NP2PTOT Movement Disorder Society Unified Parkinson’s Disease Rating Scale Part 2 (total score), ESS_tot Epworth Sleepiness Scale (total score), MCATOT Montreal Cognitive Assessment (total score), STAI_tot State-Trait Anxiety Inventory (total score), REM_tot REM Sleep Behavior Questionnaire (total score), GDS_tot Geriatric Depression Scale (total score), df degrees of freedom.

Lastly, we assessed whether the immune profile is associated with clinical measures of disease activity. Using seven clinical scores and two DaT imaging variables (Table 3), we assessed the relationship between PD symptomology and the proportions of immune cells (Fig. 5). At the baseline visit, we identified that patients in the highest quartile of sleep disturbances had lower proportions of CD8mem (${\hat{\beta }}_{Z}\,$= -0.24, *p* = 0.038) and Bmem (${\hat{\beta }}_{Z}\,$= -0.045, *p* = 0.015) compared to those in the lowest quartile (Fig. 5a). We identified decreased proportions of CD4nv (${\hat{\beta }}_{Z}\,$= -0.27, *p* = 0.043) when comparing patients in the highest quartile for cognitive dysfunction (low MoCA) to those in the lowest quartile (Fig. 5b). We also identified an association between Bmem proportions and scores on STAI (${\hat{\beta }}_{Z}$ = 0.0063, *p* = 0.017) and GDS (${\hat{\beta }}_{Z}\,$= 0.048, *p* = 0.017) (Supplementary Fig. 4), but no other associations were found at baseline (Supplementary Fig. 4, Supplementary Fig. 5).

**Table 3:** Details of the subset of clinical evaluation data used in this study.

Abbreviation	Name	Symptom(s) measured	Correlation between score and symptom severity	Score Range	Validated Score Cutoffs	Reference
MDS-UPDRS1	Movement Disorder Society Unified Parkinson’s Disease Rating Scale Part 1 (total score)	non-motor experiences of daily living	positive	0-52	>10 for moderate; >22 for severe	^117^
MDS-UPDRS2	Movement Disorder Society Unified Parkinson’s Disease Rating Scale Part 2 (total score)	motor experiences of daily living	positive	0-52	>12 for moderate; >30 for severe	^117^
ESS	Epworth Sleepiness Scale (total score)	excessive sleepiness	positive	0-24	>10	^118,119^
MoCA	Montreal Cognitive Assessment (total score)	mild cognitive impairment	negative	0-30	<26	^120^
STAI	State-Trait Anxiety Inventory (total score)	anxiety	positive	20-80	>38 for moderate; >44 for severe	^121,122^
REM	REM Sleep Behavior Questionnaire (total score)	sleep disturbances	positive	0-13	>5	^123^
GDS	Geriatric Depression Scale (total score)	depression	positive	0-15	>8 for moderate; >11 for severe	^124^
SBR	DaT SPECT: Striatal Binding Ratio	Loss of presynaptic DA neurons	negative			^125^
AsI	DaT SPECT: Asymmetry Index	Asymmetry of left and right brain regions	positive			^126^

Selected measures were utilized to capture broad areas of PD symptomology such as motor, nonmotor, psychological, and cognitive deficits.

We performed k-means clustering on the clinical variable data at baseline and at the year 2 timepoint which cluster the patients into two groups (Fig. 5c, d). Group 2 exhibited worse clinical scores across all variables; thus, we interpreted that the clustering split patients into “more symptomatic” (Group 2) and “less symptomatic” (Group 1). We compared the immune cell proportions of Group 2 to Group 1 at baseline (Fig. 5e) and at the year 2 time point (Fig. 5f). Although these findings were not statistically significant, we observed that more symptomatic patients had higher proportions of Bmem at baseline (${\hat{\beta }}_{Z}$ = 0.18, *p* = 0.11), increased proportions of Neu at year 2 (${\hat{\beta }}_{Z}$ = 1.27, *p* = 0.32), and decreased proportions of Mono at year 2 (${\hat{\beta }}_{Z}$ = -0.55, *p* = 0.072). We tested for a potential interaction between cluster group and time to determine if the rate of change of immune cells in each group was unique to each cluster. For all 11 cell types, the test was significant: Bas (p = 6.9E-04), Eos (*p* = 0.0035), Neu (*p* = 0.022), Mono (*p* = 0.0048), Bmem (*p* = 0.0016), Bnv (*p* = 0.0042), NK (*p* = 0.0043), CD4nv (*p* = 0.0040), CD8nv (*p* = 9.9E-04), CD8mem (*p* = 0.022) and CD4mem (*p* = 0.010).

### Comparing the DNAm profiles of PD and Prod groups to HC

**Fig. 6:**
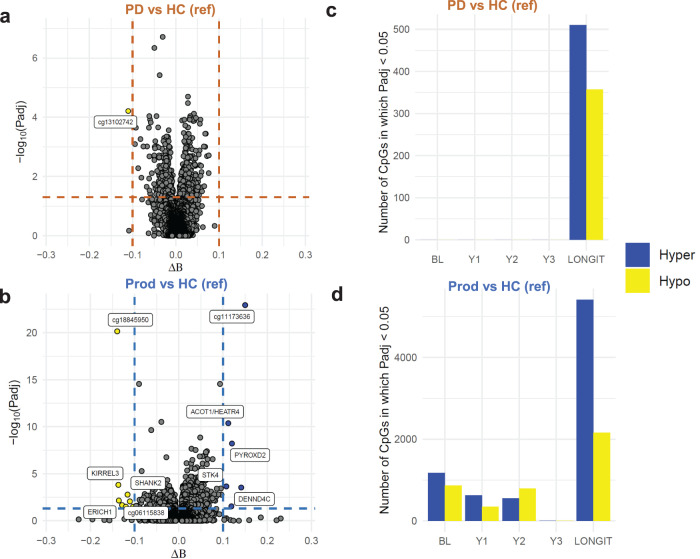
Cross-sectional and longitudinal EWAS analyses in the PPMI DNAm data. In (**a**, **c**), PD patients are compared to HC while in (**b**, **d**), prodromal PD patients are compared to HC. **a**, **b** show volcano plots highlighting differentially methylated CpGs (DMCs). False discovery rate was controlled at 5% using the BH procedure to generate adjusted *p* values (*p* values generated from empirical Bayes moderated regression model *t* test). **c**, **d** show bar plots comparing the DMCs identified in each cross-sectional model compared to the longitudinal model. The input to each EWAS analyses was the top 100,000 CpGs with the largest Δ*β*.

We performed cross-sectional and longitudinal epigenome-wide association studies (EWAS) to identify which CpGs were differentially methylated between PD and HC and between Prod and HC (Fig. 6). These models were adjusted for age at the baseline visit, sex, batch effects, and Neu, Mono, Bnv, CD4mem, and Eos proportions. A differentially methylated CpG (DMC) is defined when the average difference in methylation level (Δβ) is greater than |0.1| and the adjusted p-value to maintain a false discovery rate of 5% is still significant.

We did not identify any DMCs between PD and HC in the cross-sectional models; however, we found 1 DMC in the longitudinal model (cg13102742, Fig. 6a). Considering only the number of statistically significant CpGs, regardless of the Δβ, we still observe no DMCs in the cross-sectional models but many DMCs in the longitudinal model (Fig. 6c).

Surprisingly, we observed much more epigenetic dysregulation in the Prod group. Our longitudinal model identified 13 hypermethylated and 15 hypomethylated DMCs between Prod and HC (Fig. 6b, Supplementary Figs. 6-8). Hypermethylated DMCs were associated with *PLEKHA1*, *VASN*, *CORO7*, *DENND4C*, *NUP93*, *PYROXD2*, *L3HYPDH*, *ACOT1*, *HEATR4*, *STK4*. Hypomethylated DMCs were associated with *SPATA4*, *LENG8-AS1*, *LENG8*, *BHLHE40*, *GLIPR2*, *EPB41L5*, *PKD1L2*, *KIRREL3*, *ERICH1*, *RAD51B*, *LSG1*, *SHANK2*, and *PCNX1*. We identified DMCs in each cross-sectional model but vastly more in the longitudinal model (Fig. 6d). Most of the identified DMCs lie within 50 base-pairs of previously identified lowly-penentrant SNPs (Supplementary Table 2).

Lastly, we performed similar EWAS analyses comparing the PD group to the Prod group (Supplementary Fig. 9). We identified that two CpGs (cg18845950 and a CpG near *SHANK2*) were hypermethylated in the PD patients. These two CpGs were hypomethylated when comparing Prod to HC suggesting that these molecular alterations are indeed specific to the Prod group samples.

### PD risk factors have varying effects on the immune cell proportions present in the blood

**Fig. 7:**
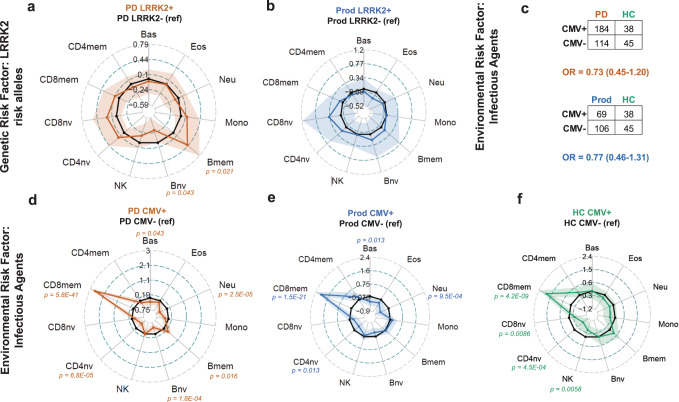
Genetic and infectious risk factors of PD are associated with perturbations to the DNAm derived immune profile. All radar plots represent variance normalized mean difference in cell proportion with 95% confidence intervals. **a**, **b** compare the immune cell proportions between LRRK2 risk allele positive patients to LRRK2 risk allele negative patients, within the PD group (**a**) or Prod group (**b**). **c**–**f** Is in reference to DNAm derived CMV serostatus. **c** shows the number of predicted seropositive and seronegative patients in each group. **d**–**f** compare the immune cell proportions in CMV seropositive to CMV seronegative participants in each group. Models were adjusted for age, sex, and batch variables. Only *p* values <0.05 are denoted (regression model *t* test; df = 291 for (**a**); df = 169 for (**b**); df = 292 for (**d**); df = 169 for (**e**); df = 78 for (**f**)). HC healthy control group, PD Parkinson’s disease group, Prod prodromal Parkinson’s disease group, Bas basophils, Eos eosinophils, Neu neutrophils, Bnv B-cells naïve, Bmem B-cells memory, CD4nv helper CD4^+^ T-cells naïve, CD4mem helper CD4^+^ T-cells memory, Treg CD4^+^ T regulatory cells, CD8nv cytotoxic CD8^+^ T-cells naïve, CD8mem cytotoxic CD8^+^ T-cells memory, Mono monocytes, NK natural killer cells, df degrees of freedom.

Previous findings have shown a genetic influence on the peripheral immune cell proportions in PD patients^23,59^. Using the baseline sample data, we compared the immune cell composition of *LRRK2* risk allele positive (*LRRK2*^*+*^) PD patients with the major allele of *LRRK2* (*LRRK2*^*-*^) (Fig. 7a). We identified higher proportions of Bmem (${\hat{\beta }}_{Z}$ = 0.29, *p* = 0.021) but lower proportions of Bnv (${\hat{\beta }}_{Z}\,$= -0.40, *p* = 0.043) in *LRRK2*^*+*^ PD patients. Similarly, we repeated this analysis with LRRK2+ and LRRK2- Prod patients but did not identify these differences (Fig. 7b). We also performed this analysis for GBA risk allele patients. However, as anticipated we were underpowered to test for this, given the low number of GBA+ patients (Supplementary Fig. 10).

We also assessed how environmental risk factors, such as viral infections, contributed to changes in the immune cell composition of the blood^60^. We utilized a DNAm-based prediction model to predict cytomegalovirus (CMV) seropositivity. We did not identify an association between CMV seropositivity and having PD (Fig. 7c). However, we observed very large differences in immune cell composition between individuals who were predicted to be CMV seropositive (CMV + ) and those who were predicted to be seronegative (CMV-) (Fig. 7d–f). Across all three patient groups, we find CMV+ subjects to have elevated CD8mem (HC ${\hat{\beta }}_{Z}\,$= 6.1, *p* = 4.2E-9; Prod ${\hat{\beta }}_{Z}$ = 6.7, *p* = 1.5E-21; PD ${\hat{\beta }}_{Z}$ = 7.6, *p* = 5.8E-41) and decreased CD4nv (HC ${\hat{\beta }}_{Z}$ = -2.3, p = 4.5E-4; Prod ${\hat{\beta }}_{Z}$ = -1.5, p = 0.013; PD ${\hat{\beta }}_{Z}$ = -1.5, *p* = 6.8E-5). We identified a decrease in Neu in CMV + PD (${\hat{\beta }}_{Z}$ = -4.3, *p* = 2.5E-5) and Prod (${\hat{\beta }}_{Z}\,$= 0.29, *p* = 0.021) subjects that is not observed in CMV + HC. Similarly, we saw only changes in Bnv (${\hat{\beta }}_{Z}\,$= -0.60, *p* = 1.8E-4) and Bmem (${\hat{\beta }}_{Z}$ = 0.25, *p* = 0.048) proportions in the CMV + PD subjects but not the other groups. Lastly, a decrease in NK cells in CMV + HC (${\hat{\beta }}_{Z}$ = -1.3, *p* = 0.058) was not observed in CMV + PD or CMV+ Prod subjects. Together these data support that both genetic and environmental risk factors of PD also disrupt the proportions of peripheral immune cells in the blood of PD and Prod patients.

**Fig. 8:**
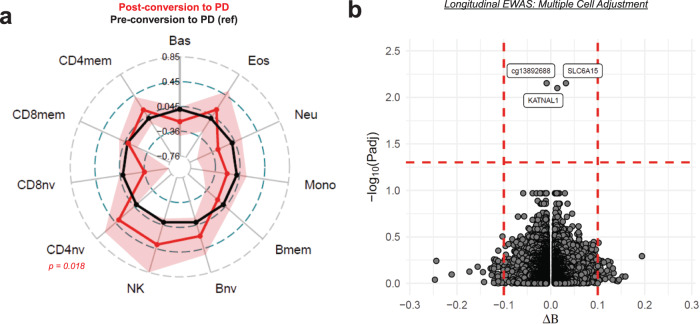
Analyzing the DNAm data from Prod patients that converted to PD during study follow-up. **a** compares the samples from patients after their clinical diagnosis of PD to those before their diagnosis of PD. The values represented are variance normalized mean differences in cell proportion with 95% confidence intervals. *P* values were generated from regression model *t* tests; df = 71.5). **b** shows the EWAS comparing the same set of samples. (*p* values generated from empirical Bayes moderated regression model *t* test) and the false discovery rate was controlled at 5%. The input to each EWAS analyses was the top 100,000 CpGs with the largest Δ*β*. Bas basophils, Eos eosinophils, Neu neutrophils, Bnv B-cells naïve, Bmem B-cells memory, CD4nv helper CD4^+^ T-cells naïve, CD4mem helper CD4^+^ T-cells memory, Treg CD4^+^ T regulatory cells, CD8nv cytotoxic CD8^+^ T-cells naïve, CD8mem cytotoxic CD8^+^ T-cells memory, Mono monocytes, NK natural killer cells, df degrees of freedom.

For our final analysis, we wanted to assess if there were changes in immune cell composition in the Prod subjects that converted to PD. For a small subset of patients, the PPMI collected DNAm data on patients before and after their PD diagnosis (*n* = 20, Supplementary Fig. 11). We compared their pre and post-conversion samples and identified an increase in CD4nv post-conversion (Fig. 8a). We also performed an EWAS analysis to identify possible DMCs associated with conversion to PD (Fig. 8b). Although no DMC surpassed our Δβ threshold, the model identified three statistically significant DMCs (cg13892688, *SLC6A15*, *KATNAL1*).

## Discussion

From longitudinally collected blood DNAm data in the PPMI database, we performed an immune profiling study on clinically defined PD patients, prodromal PD patients and matched neurologically healthy participants. We sought to validate others’ findings about the relative quantities of peripheral immune cells in PD and determine at which point along the disease course these immunological perturbations occur.

We identified significant perturbations to the proportions of innate immune cells in well-established PD. Comparing PD to HC, we found an increase in neutrophils and a decrease in monocytes and eosinophils. In PD patients, there is an increase in neutrophils and monocytes over time, with a consistent trend in the Prod group as well. We observed a slight decrease in monocytes in more symptomatic PD patients. Lastly, although we observed changes in the innate immune cells at the baseline PD timepoint, there is not a strong change in the innate immune cell compartment as patients transition from prodromal PD to PD, except for a slight increase in NK cells.

There are conflicting results on whether the proportions of neutrophils change in the blood of PD patients, with some papers stating no changes to healthy controls, while others report higher neutrophil proportions or a higher neutrophil to lymphocyte ratio (NLR)^23,50,61–63^. Neutrophils are notoriously fragile and can easily be underrepresented in single-cell RNA sequencing or flow cytometry if the sample processing is not correct, which may explain why this result is mixed across studies^64–67^. Here, we leverage DNAm cell deconvolution strategies to analyze neutrophils in which we identified increases in the proportions of neutrophils in PD patients. In Alzheimer’s disease (AD), it is well established that the release of neutrophil extracellular traps (NET) in the vasculature of the brain can increase the permeability of the blood-brain barrier which facilitates chronic inflammation^68^. Our results support the notion that these cells may be involved in similar pathogenic mechanisms across chronic neuroinflammatory diseases including PD.

In concordance with others’ findings, we also reproduced a decrease in monocytes in PD patients^22–24^. It was interesting as well that we found this effect to be stronger and more correlated to symptom severity at later rather than earlier time points, which is supported in the literature by known trends in activity and phenotype as PD progresses^69^. Pigment-laden macrophages are observed in the degenerating mesencephalon in PD but it remains unclear to what degree peripheral monocytes contribute to this population typically thought to arise from CNS resident microglia. A decrease in circulating monocytes may result from increases in monocyte diapedesis into the CNS in response to tissue damage. The analysis described here provides an important starting point for studies designed to further subset peripheral immune cells like monocytes to contextualize their states concerning CNS damage.

Taking these findings together, we conclude that perturbations to the innate cell compartment manifest when PD is highly symptomatic and developed, whereas at very early stages of the disease, such as during the prodromal stage or very close to diagnosis, we see smaller changes in the relative proportions of innate immune cells.

We found that perturbations to the adaptive immune landscape occur at slightly different periods compared to innate immune perturbations. We identified a strong decrease in CD4^+^ memory T cells and naive B cells in clinically defined and prodromal PD. In PD patients, we see a significant decrease in many adaptive immune cell populations over time, including all naive lymphocytes and memory T cells. In the prodromal group, there is a significant decrease in the naïve lymphocytes but not a significant trend in the memory compartments. Symptomology changes correlated more with proportions of adaptive immune cells than innate cells. For instance, proportions of all memory lymphocyte populations were associated with REM sleep disturbances, which matches previous findings about the relationships of peripheral T cell phenotypes associated with this phenotype^70^. In addition, higher proportions of CD4^+^ naive cells were identified in patients with less cognitive decline and we found a slight increase in B memory cells in those who were more symptomatic at baseline. B memory cells were also higher in PD patients with more symptoms of depression and anxiety. Strikingly, when we compared samples of prodromal PD patients before and after their transition to clinically defined PD, we see a significant increase in CD4^+^ naive cells after diagnosis.

Across multiple studies, there is a decrease in T cells in the periphery, primarily naive T cell and CD4^+^ T cell populations^23,50,54,71,72^. Some studies also report a decrease in naive B cells and an increase in memory B cells within the peripheral blood, which matches the results of our analysis as well^54,73^. One single-cell RNA sequencing study identified an increase in isotype-switched (IgG and IgA) proportions in PD blood further supporting our results that the B cell compartment in PD patients is more antigen-experienced and shifted to more mature B cell phenotypes^74^.

Together, these data suggest that the perturbations to the peripheral adaptive immune profile are linked with early or acute disease events in PD. We believe that in “preclinical” PD, an adaptive immune response may be initiated against a range of antigens, possibly emanating from the gut, stress related to environmental insult, to CNS antigens related to early-stage neurodegenerative processes. Our findings are consistent with “systemic” PD, demonstrating that levels of various T and B cell populations correlated with non-motor symptoms such as sleep disturbances and cognitive ability. We observe another increase in the CD4^+^ naive T cell levels in patients right after they transition to a clinical diagnosis of PD. We hypothesize that the initial presentation of motor symptoms is associated with increases in neurodegenerative antigens and possibly the dissemination of protienopathic stressors such as seeding competent alpha synuclein modulating T cell state and leading to more naive T cells responding to or being recruited to the CNS^34^.

However, as degeneration progresses and there is sustained CNS inflammation, we observe larger changes to the innate immune system. In clinically defined PD patients, there is a significant decrease in monocyte and increase in neutrophil proportions compared to healthy controls. However, over time the proportions of both are increasing. For monocytes, we suspect that the decreased level in the periphery compared to controls may be due to an increased infiltration into the CNS, consistent with work done in rodents^75,76^. The levels of monocytes may be increasing over time as more CNS damage takes place and recruits more innate immune cells. The increase in neutrophils we suspect are involved in opening of the BBB to recruit more immune cells to the CNS, as others have described BBB instability in PD patients^77^. Similarly, BBB instability has been associated with neutrophil activity in multiple sclerosis (MS) and Alzheimer’s disease^78,79^. As the levels of monocytes and neutrophils are increasing in PD patients over time, we see a decrease in many lymphocyte population levels over time. Compared to healthy controls, PD patients exhibit a decrease in CD4^+^ memory cells and naive B cells. Because the levels of these cells are lower and they are decreasing over time, it can be interpreted in two different manners: either (1) that there is migration of these cells to the CNS, causing a decrease of their levels in the periphery or (2) there is a dampening of the adaptive immune response as a protective mechanism to mitigate the risk of CNS autoimmunity. Current hypotheses pose that chronic inflammatory signaling can cause a shift from adaptive to innate immunity as the predominating inflammatory mechanism during the progression of CNS disorders^80–87^. Further functional studies of these cell types from early PD patients are required to test these hypotheses.

We performed longitudinal EWAS analyses using (1) a cross-sectional EWAS analysis at each major study timepoint or (2) a longitudinal EWAS analysis by adding a random effect into the model, which will account for the longitudinally in the dataset. We found that in all of our comparisons (PD vs. HC, Prod vs. HC, and PD vs. Prod), more DMCs were called in our longitudinal models compared to the cross-sectional models. We believe that in the longitudinal modeling approach, within-patient variation is better accounted for via the random effect, and thus this increases the power of hypothesis testing.

When comparing PD vs. HC, we found a single differentially methylated CpG, cg13102742, previously associated with schizophrenia^88^. We saw much more of an epigenetic shift when comparing Prod vs. HC. When visualizing the distribution of DNAm values at these positions, we noticed methylation patterns that may be indicative of underlying genetic variation, since we observe a trimodal distribution centered at 0, 0.5, and 1 for many of these DMCs and that these DMCs lie close to lowly-penetrant SNPs. Our findings show that the DNA methylome seems to be more deviated from HC in the Prod group compared to the PD group. This may suggest that the DNAm perturbations are more associated with earlier disease events, in other words, the initial immune responses to alpha-synuclein.

There has been a strong body of research investigating the genetic and inheritable risk factors for developing PD identifying risk variants to glucocerebrosidase (*GBA*) and leucine-rich repeat kinase 2 (*LRRK2*) among others^89,90^. *LRRK2* variants with pathogenic associations with PD account for 5–13% of familial PD cases and 1–5% of sporadic PD^91^. Variation in this protein function is thought to disrupt kinase cascades and alpha-synuclein clearance in the brain^91^. Interestingly, *LRRK2* may also act on the neuro-immune axis in PD and other chronic inflammatory diseases^92–94^. Others have shown a potential interaction between that the *LRRK2* genotype and PD case status when looking at the proportions of immune cells^23^. We show that *LRRK2*^*+*^ PD patients had lower proportions of naive B cells and higher proportions of memory B cells. It has been shown that *LRRK2* is expressed in B cells and mice lacking *LRRK2* have a more naive B cell repertoire^95^. Because this B cell trend was not observed in the Prod cohort, we hypothesize that the inflammatory-mediated genetic risk of *LRRK2* variants may affect the chronic immune responses in defined PD, rather than the first presence of neuronal debris or alpha-synuclein. We repeated this analysis comparing *GBA* positive and negative PD patients, however, in this dataset only 20 patients were *GBA* allele positive, which greatly limited our statistical power. Although the trends were not significant, we did observe many of the trends to be in the same direction as the *LRRK2*^*+*^ vs. *LRRK2*^*-*^ comparison. Similarly, others have stated an effect of PD-related GBA mutations on B cell biology^96^, thus, more research is needed to further understand how this peripheral immune activity mediates the effect of these genetic alleles on PD risk.

Many PD risk factors are related to environmental exposures such as infections. Since there may be an association between PD and viral infections, including herpes viruses^97,98^, we wished to test this association in the PPMI data by predicting cytomegalovirus (CMV) seroprevalence from the DNAm data^99^. Although the relationship between seroprevalence for CMV and PD risk is not entirely clear^60,100^, recent literature has shown there may be disrupted T cell and myeloid populations in CMV seropositive PD patients^101,102^. Firstly, we did not identify a significant association between CMV seroprevalence and having PD. However, CMV infection has been tied to various diseases; thus, this our estimate is susceptible to selection bias by over-selecting HC who did not contract CMV or are resilient to CMV-related conditions. In our HC population, we found CMV positive participants had higher proportions of CD8^+^ memory T cells, with decreases in NK cells, CD8^+^, and CD4^+^ naive T cells. We found these perturbations in the other groups; however, the effect was weakest in the HC group and stronger in PD patients. However, CMV positive PD and Prod patients have lower proportions of neutrophils compared to CMV negative patients and this was not observed in the HC group. Furthermore, the PD CMV positive patients exhibited a decrease in naive B cells and an increase in B memory cells. These alterations were in the same direction as what was observed with the effect LRRK2 genotype on the peripheral immune cell proportions. Together these data suggest that the way the genetic and environmental PD risk factors affect the peripheral immune system may follow overlapping mechanisms and that combinations of genetic and environmental risk may be synergistic concerning the effects on inflammation. Our findings that B cell perturbations are found in the Prod population and are associated with genetic and infectious risk factors of PD provide a rationale for the further study of B cells in early PD.

Although this study is in line with previous literature, there are certain limitations. Firstly, we anticipate residual confounding in our results due to medication use and the presence of other comorbidities. Although all patients were medication naive at baseline, some patients did go on to receive treatment during follow-up. Secondly, our analyses consider proportions of cell types rather than actual counts. Third, we are limited by the availability of deconvolution libraries to resolve certain subsets of leukocytes, such as immature from mature neutrophils or helper T-cell subtypes. As reference libraries for these cell types are developed, these analyses can be repeated to gain said resolution. Fourth, in our longitudinal analyses, we used the patient visit ID coding system to determine the number of months away from baseline the sample was taken place. This “binning” of the data will introduce a slight nondifferential misclassification bias in the data concerning the true timing of the sample. Fifth, we observed a batch effect that could not be explained by known technical or biological variables. Although we adjust for this in our analysis and performed sensitivity analyses, it remains unclear as to what is the source of this variation. Lastly, the direction of our hypothesis testing was to test if there was an effect of having PD on the proportion of a particular immune cell. Thus, we are assuming that having PD is causal to the change in immune cell proportion. There is potential for reverse causality, in which the change in immune cell proportion is casual to having PD. Until we understand more about the underlying biology of inflammation and neuroinflammation in PD, the direction of this hypothesis testing is left up to debate.

By utilizing blood DNAm from the PPMI cohort, we were able to employ cellular deconvolution to predict the proportions of immune cells and conduct a longitudinal immune profiling study. We established that in prodromal PD, the immune landscape is significantly different in age and sex-matched healthy individuals. This suggests that the peripheral immune system is responding to the earliest presence of CNS antigens (alpha-synuclein and/or CNS debris). Our findings suggest that the change in the peripheral adaptive immune profile is altered with earlier disease events. However, as the patients accrue more clinical burden and neurodegeneration accelerates, we observe dynamic shifts of innate and memory populations, which may be representative of a shift toward a chronic inflammatory state.

## Methods

### PPMI patient selection and data collection

For detailed patient recruitment and study protocols, please visit the PPMI consortium publications^56,57^, the PPMI website (https://www.ppmi-info.org/study-design/research-documents-and-sops), and ClinicalTrials.gov (NCT01141023, registered June 10, 2010). In brief, all participants gave informed consent before enrollment. Participants were organized into one of three groups (Table 1): Parkinson’s disease (PD), healthy control (HC), or prodromal PD (Prod). The PD group consists of people with clinically defined PD^4^. Individuals were excluded if they had received their diagnosis more than two years before screening or received, or expected to receive, treatment for PD (levodopa, dopamine agonists, MAO-B inhibitors, or amantadine) in the initial six months of follow-up. The HC group comprises age- and sex-matched individuals without significant neurological disorders and naive to PD-related medications. Lastly, the Prod group includes people with an elevated risk of progressing to PD. Individuals were excluded from this group if they met the criteria of clinically defined PD. However, a combination of symptomology (hyposmia or REM sleep behavior disorder) or genetic background (first-degree biological relative or specific gene variants) excluded these individuals from being in the HC group (Table 2). The consensus committee determined the assignment of each patient to a group through PPMI. All three groups of patients had a baseline sample (BL) in which basic demographic information and medical history were recorded. These patients were followed longitudinally with 1-4 visits per year for clinical evaluation and biospecimen collection. The following data were included in our analyses:

#### Whole blood DNAm data

Genomic DNA from blood collected at study visits was isolated (AutoGen Flex DNA extraction), quantified (Qubit 2.0 Fluorometer), bisulfite converted (Zymo EZ-96 DNA Methylation Kit), and loaded onto Infinium MethylationEPIC BeadChip arrays (Illumina, v1.0). The arrays were read with the Illumina iScan System to develop raw .idat files which contain DNAm data for over 850,000 CpG sites in the human genome.

#### Dopamine Transporter (DaTSCAN) Single-photon emission computerized tomography (SPECT)

The DaTScan SPECT imaging^103^ was done according to the PPMI imaging protocol. This imaging modality allows for visualizing DA presynaptic neuron loss in the striatum. At least two nuclear medicine experts analyzed all images for interpretation. Raw data were reconstructed and processed according to the stated methods for calculating the striatal binding ratio (SBR). In brief, regions of interest were placed on 9-slice averaged images to select the left and right putamen and caudate. The occipital cortex was used as a reference. The SBR was calculated as the ratio of the signal intensity of the target region to the reference region minus one. The asymmetry index (AsI) is calculated as [(R-L)/(R + L)]*100 where R and L represent the SBR of the left and right putamen or caudate (Table 3).

#### Clinical evaluations

For this analysis, we used data on seven clinical exams (MDS-UPDRS1, MDS-UPDRS2, ESS, MoCA, STAI, REM, and GDS) to assess possible relationships with different sets of PD-related symptoms (Table 3).

### DNAm data preprocessing

Data preprocessing and analysis were done in R Studio (v4.2). All available DNAm data as of July 2022 were downloaded from the PPMI web interface in raw .idat format. Patient data were matched to available DNAm data. Patient visits annotated to multiple arrays was excluded. Additionally, if any sample had missing sex or age information, it was also excluded. The remaining data were analyzed using a combination of functions from various R packages, including *minfi* [v1.42.0]^104,105^, *sesame* [v1.14.2]^106^, *ENmix* [v1.32.0]^107,108^, and *ewastools* [v1.7]^109^. We utilized normal-exponential out-of-band correction with linear dye-bias normalization to derive a beta value for every array position for every sample^110^.

### DNAm data quality control and filtering

We first employed a sample-wise filtering approach to determine each array’s quality and remove data that fails quality control assessments. Each sample had to pass three criteria to be included in the analysis: (1) the median beta value of the sample had to lie within three inter-quartile ranges of the set of all median beta values in the study, (2) the predicted sex, as estimated by the *minfi* package, matches the reported sex, (3) all quality control probe signals are within the appropriate regions as identified by the *ENmix and ewastools* software. The number of samples and their reason for exclusion are listed in Supplementary Table 1.

Next, we employed a probe-wise filtering approach to remove poor-quality probe data within each sample. Detection p-values were used to determine if the intensity signal for each probe was significantly above the background control probes (*p* < 0.05). If the probe fails, its beta value was masked as missing. In addition, probes located on the X or Y chromosome, near single nucleotide polymorphisms (SNPs), or in regions of known array cross-reactivity were masked, allowing for a final set of 743,840 CpGs of data to remain in the study for analysis. For each CpG, a β value was calculated, representing the methylation level at that position.

### Calculation of DNAm-derived variables

#### Blood immune cell proportions

The blood immune cell fractions were calculated using the FlowSorted.BloodExtended.EPIC software^42^. These values were then adjusted for their limit of detection (LoD) values as previously described^111^. Any value below the cell type-specific LoD was replaced by the LoD/$\surd 2$. After replacing the LoD, the remaining cell types were scaled, so the sum of all cell proportions was constrained to 1.

#### Cytomegalovirus (CMV) seropositivity prediction

CMV seropositivity was calculated with a previously published elastic net prediction model with the output delivered as a binary seropositive or seronegative for CMV^99^.

### Statistical analysis: comparing immune cell compositions

We utilized linear regression models to test the effect of a binary variable, ${X}_{Z}$, on the composition of immune cells at a single cross-sectional timepoint. Let ${y}_{i}$ represent the proportion of cell type $i$. For every cell type, the cross-sectional regression model at a given timepoint is represented as Eq. I where $\alpha$ is the intercept term, $\varepsilon$ is the error term, and $X$ is the covariate matrix, consisting of a binary variable for sex, a continuous variable for the age of the patient at their baseline visit, and two binary batch effect variables. An estimate for ${\hat{\beta }}_{{Z}_{i}}$ and 95% confidence intervals were calculated for each cell type and plotted on the radar plots. P-values were generated for each estimate by performing a regression model t-test to determine if estimate was significantly nonzero. For the variance-normalized radar plots, the estimate and confidence intervals for ${\hat{\beta }}_{{Z}_{i}}$ was divided by the standard deviation of ${y}_{i}$ in the reference group (Eq. II). We adapted this approach for longitudinal data by introducing a random effects term to Eq. I by adding a random effect for each patient (${u}_{{subject}}$) and time as a continuous fixed effect (Eq. III). To identify if there was an interaction between time and ${X}_{Z}$ for each cell type, we performed likelihood ratio tests using the *lmtest* and *lmerTest* software, comparing the models generated by Eq. III and Eq. IV to test the hypothesis that adding the interaction better fits the data^112,113^.

I $${y}_{i}={\alpha }_{i}+{\hat{\beta }}_{{Z}_{i}}{X}_{z}+{\hat{\beta }}_{{{sex}}_{i}}{X}_{{sex}}+{\hat{\beta }}_{{{age}}_{i}}{X}_{{age}}+{\hat{\beta }}_{{{batch}1}_{i}}{X}_{{batch}1}+{\hat{\beta }}_{{{batch}2}_{i}}{X}_{{batch}2}+\varepsilon$$

II $$\triangle {y}_{i}={\hat{\beta }}_{{Z}_{i}}/{y}_{i}$$

III $${y}_{i}={\alpha }_{i}+{\hat{\beta }}_{{Z}_{i}}{X}_{z}+{\hat{\beta }}_{{{sex}}_{i}}{X}_{{sex}}+{\hat{\beta }}_{{{age}}_{i}}{X}_{{age}}+{\hat{\beta }}_{{{batch}1}_{i}}{X}_{{batch}1}+{\hat{\beta }}_{{{batch}2}_{i}}{X}_{{batch}2}+{\hat{\beta }}_{{{time}}_{i}}{X}_{{time}}+{u}_{{subject},i}+\varepsilon$$

IV $${y}_{i}={\alpha }_{i}+{\hat{\beta }}_{{Z}_{i}}{X}_{z}+{\hat{\beta }}_{{{sex}}_{i}}{X}_{{sex}}+{\hat{\beta }}_{{{age}}_{i}}{X}_{{age}}+{\hat{\beta }}_{{{batch}1}_{i}}{X}_{{batch}1}+{\hat{\beta }}_{{{batch}2}_{i}}{X}_{{batch}2}+{\hat{\beta }}_{{{time}}_{i}}{X}_{{time}}+{\hat{\beta }}_{{{interaction}}_{i}}{X}_{Z}{X}_{{time}}+{u}_{{subject},i}+\varepsilon$$

### Statistical Analysis: K-means clustering of clinical data

We performed k-means clustering using the *cluster* software^114^, including the following clinical variables: MDS-UPDRS1, MDS-UPDRS2, ESS, MoCA, GDS, STAI, REM, putamen AsI, and caudate AsI. Values were mean-centered and scaled before clustering. Decisions about the optimal number of clusters to divide the data were determined using the *fviz_nbclust* and *clusGap* visualizations.

### Statistical analysis: longitudinal epigenome-wide association studies (EWAS)

EWAS analysis was done using the *minfi* and *limma* software^115^. To identify differentially methylated CpGs (DMCs) between two groups, we utilized the same regression approach as in Eq. I, but instead ${y}_{i}$ represents the DNAm level at CpG $i$. DNAm proportions (β values) were converted to M-values by computing the logit of the β values in base 2. We subset the EWAS analyses to the top 100,000 CpGs with the largest mean difference in β values (Δβ) between the two groups. An empirical Bayes method was used to normalize CpG-wise residual variance. P-values for each CpG were generated using regression model t-tests to determine if the coefficient is nonzero. P-values were adjusted for multiple hypothesis testing using the Benjamini-Hochberg procedure to maintain a false discovery rate of 5%. To perform a longitudinal EWAS analysis, we utilize the *duplicateCorrelation* functionality in the *limma* software to produce models equivalent to Eq. II for differential methylation testing, including the random effect to adjust for repeated measurements.

### Reporting summary

Further information on research design is available in the Nature Research Reporting Summary linked to this article.

### Supplementary information

Supplementary File 1

Reporting Summary

## Data Availability

Data used in the preparation of this article were obtained from the Parkinson’s Progression Markers Initiative (PPMI) database (www.ppmi-info.org/access-data-specimens/download-data), RRID:SCR_006431. For up-to-date information on the study, visit www.ppmi-info.org.

## Code availability

All R scripts used to preprocess and analyze the data are available on our laboratory’s GitHub page (https://github.com/SalasLab/PPMI_DNAm_Analysis) and on Zenodo^116^.
