# Supplementary material for: Immunological shifts during early-stage Parkinson’s disease identified with DNA methylation data on longitudinally collected blood samples
Source: NPJ Parkinsons Dis. 2024 Jan 11;10:21. doi: 10.1038/s41531-023-00626-6 (PMC10784484; doi:10.1038/s41531-023-00626-6)

Supp Table 1: Details behind removal of certain samples for this analysis, stratified by patient group.

**Reason for sample being excluded from analysis**	**HC**	**PD**	**Prod**
Median beta value outside 3 IQR	0	0	1
> 10% masked data	0	4	12
Sex mismatched	1	13	7
Failed nonpolymorphic green control probes	0	0	2
Failed red specificity I control probes	0	1	7
Failed red staining control probes	0	2	1
Failed green staining control probes	6	19	7
**Total**	**7**	**38**	**27**

Supp Table 2: Information about CpGs called in EWAS analyses and their relationship known to SNPs.

CpG Name	Chromosome	Comparison	UCSC Gene Name	SNP ID	Distance from CpG to SNP	SNP Minor Allele Frequency	Regulatory Region	Relation to CpG Island
cg13102742	chr17	PD vs HC		rs185902692;rs533083418;rs552758573;rs563085394;rs141421426;rs9330243	44;34;26;21;19;1	0.000200;0.000200;0.000200;0.000200;0.000200;0.428571	Enhancer	Open.Sea
cg18159740	chr13	Pre vs Post Conversion	KATNAL1;KATNAL1	rs564278654;rs149417687;rs566788482	48;32;8	0.001398;0.001797;0.000599	Promoter	Island
cg00154888	chr12	Pre vs Post Conversion	SLC6A15;SLC6A15;SLC6A15	rs548104255	41	0.0002	Dual	Shore
cg13892688	chr1	Pre vs Post Conversion					Gene body	Open.Sea
cg26251192	chr14	Prod vs HC	ACOT1;HEATR4;HEATR4				Enhancer	Shore
cg21223075	chr3	Prod vs HC	BHLHE40	rs527338205;rs547190424;rs373533215	1;19;36	0.000998;0.000200;0.002596	Promoter	Island
cg00924943	chr9	Prod vs HC	DENND4C;DENND4C	rs10738534	1	0.007588	Gene body	Open.Sea
cg03885684	chr2	Prod vs HC	EPB41L5	rs532897337	1	0.0002	Dual	Island
cg14755254	chr8	Prod vs HC	ERICH1;ERICH1	rs187438779;rs576214117	13;1	0.000998;0.000200	Promoter	Island
cg00685135	chr9	Prod vs HC	GLIPR2;GLIPR2;GLIPR2;GLIPR2;GLIPR2;GLIPR2;GLIPR2;GLIPR2;GLIPR2;GLIPR2;GLIPR2				Promoter	Island
cg13799572	chr11	Prod vs HC	KIRREL3;KIRREL3;KIRREL3	rs10893527	0	0.046925	Gene body	Open.Sea
cg01543583	chr14	Prod vs HC	L3HYPDH	rs79606592;rs74924576;rs1253100	18;9;1	0.017572;0.500000;0.009385	Dual	Open.Sea
cg02746014	chr19	Prod vs HC	LENG8-AS1;LENG8				Promoter	Shore
cg02078724	chr3	Prod vs HC	LSG1	rs7646730;rs1705988	22;2	0.002596;0.035144	Gene body	Open.Sea
cg22968327	chr16	Prod vs HC	NUP93;NUP93;NUP93	rs115684231	1	0.00599	Gene body	Open.Sea
cg02907150	chr14	Prod vs HC	PCNX1	rs543994918;rs562378624;rs575629215;rs116016	35;14;6;1	0.000200;0.000200;0.001597;0.191793	Gene body	Open.Sea
cg04833938	chr16	Prod vs HC	PKD1L2;PKD1L2	rs550824632;rs34719852;rs539792118	48;22;10	0.000200;0.027157;0.000200	Promoter	Open.Sea
cg10576280	chr10	Prod vs HC	PLEKHA1	rs532849834	37	0.0002	Dual	Shore
cg26690318	chr10	Prod vs HC	PYROXD2	rs201079830;rs201098004;rs200244578;rs45523432;rs12763044;rs12763379	47;46;40;31;16;1	0.001500;0.000200;0.000200;0.418131;0.417332;0.419014	Enhancer	Open.Sea
cg18683228	chr14	Prod vs HC	RAD51B;RAD51B;RAD51B	rs11158728;rs202005616;rs561757233	1;2;24	0.003395;0.002596;0.000200	Enhancer	Open.Sea
cg09157251	chr11	Prod vs HC	SHANK2	rs182590780;rs111886587;rs1000968	11;7;2	0.001997;0.005791;0.193490	Gene body	Open.Sea
cg02833127	chr4	Prod vs HC	SPATA4;SPATA4	rs200238044	2	0.000399	Dual	Island
cg06612594	chr20	Prod vs HC	STK4	rs554784913;rs568276201;rs537151634	49;40;1	0.000200;0.000200;0.000200	Enhancer	Open.Sea
cg11523661	chr17	Prod vs HC	TBC1D16	rs200133337;rs202181903;rs201292710	42;29;19	0.000200;0.000200;0.000399	Enhancer	Shore
cg16628641	chr16	Prod vs HC	VASN;CORO7	rs541522593;rs561407155;rs530114318	22;41;46	0.000399;0.000200;0.000200	Enhancer	Open.Sea
cg11173636	chr10	Prod vs HC		rs141831322;rs16918991;rs575081695;rs544302191;rs563989248	38;30;17;14;9	0.001797;0.042133;0.000200;0.000599;0.000200	Gene body	Open.Sea
cg11787544	chr13	Prod vs HC					Intergenic	Open.Sea
cg16786756	chr4	Prod vs HC		rs549352260	6	0.0002	Promoter	Island
cg18845950	chr6	Prod vs HC		rs9264960;rs9264961;rs17192631;rs529356510;rs9264962	40;39;24;9;1	0.453075;0.453075;0.180511;0.001198;0.105631	Promoter	Island
cg00088299	chr4	Prod vs HC					Promoter	Island
cg11394338	chr5	Prod vs HC		rs372893892;rs140460605;rs543348580;rs145285831	49;31;2;1	0.000200;0.003395;0.000599;0.000200	Enhancer	Open.Sea
cg06115838	chr17	Prod vs HC		rs531081678;rs73365330	39;1	0.001797;0.006390	Enhancer	Open.Sea

Supp Fig 1: Distributions of raw proportions calculated from blood deconvolution. Any values below the red LoD line were LoD adjusted. X-axis shows cellular proportion and y-axis shows the density of samples at a given composition.

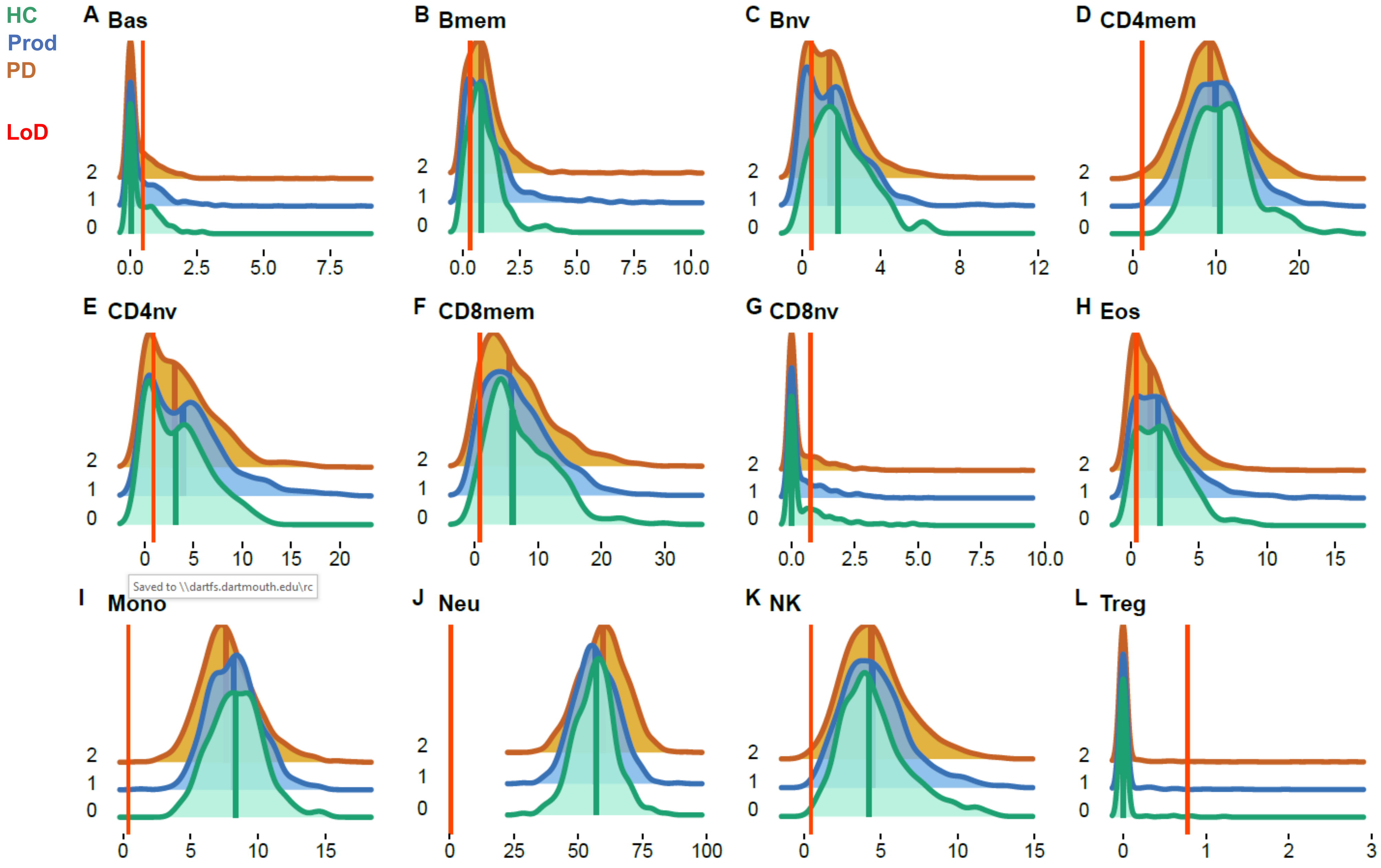

Supp Fig 2: Visualizing the two batch variables apparent in the dataset. (a) First batch variable was identified in data processing batches. (b-c) Second batch variable was identified in samples collected after 2021. The y-axis represents the DNAm β value for all panels. Boxplot bar represents the median value, boxplot hinge represents 25th and 75th quartiles and the whiskers represent 1.5 times the inter quartile range. There was low collinearity observed between these variables and variables included in regression analyses

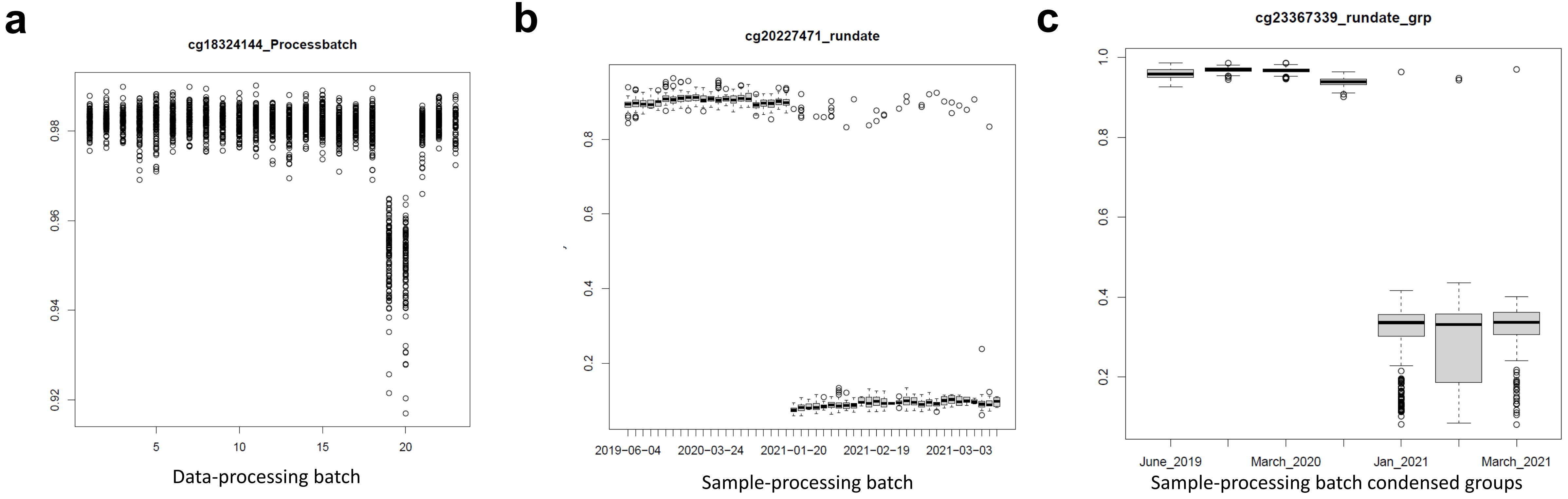

Supp Fig 3: Comparing the immune cell composition between PD and Prod groups.

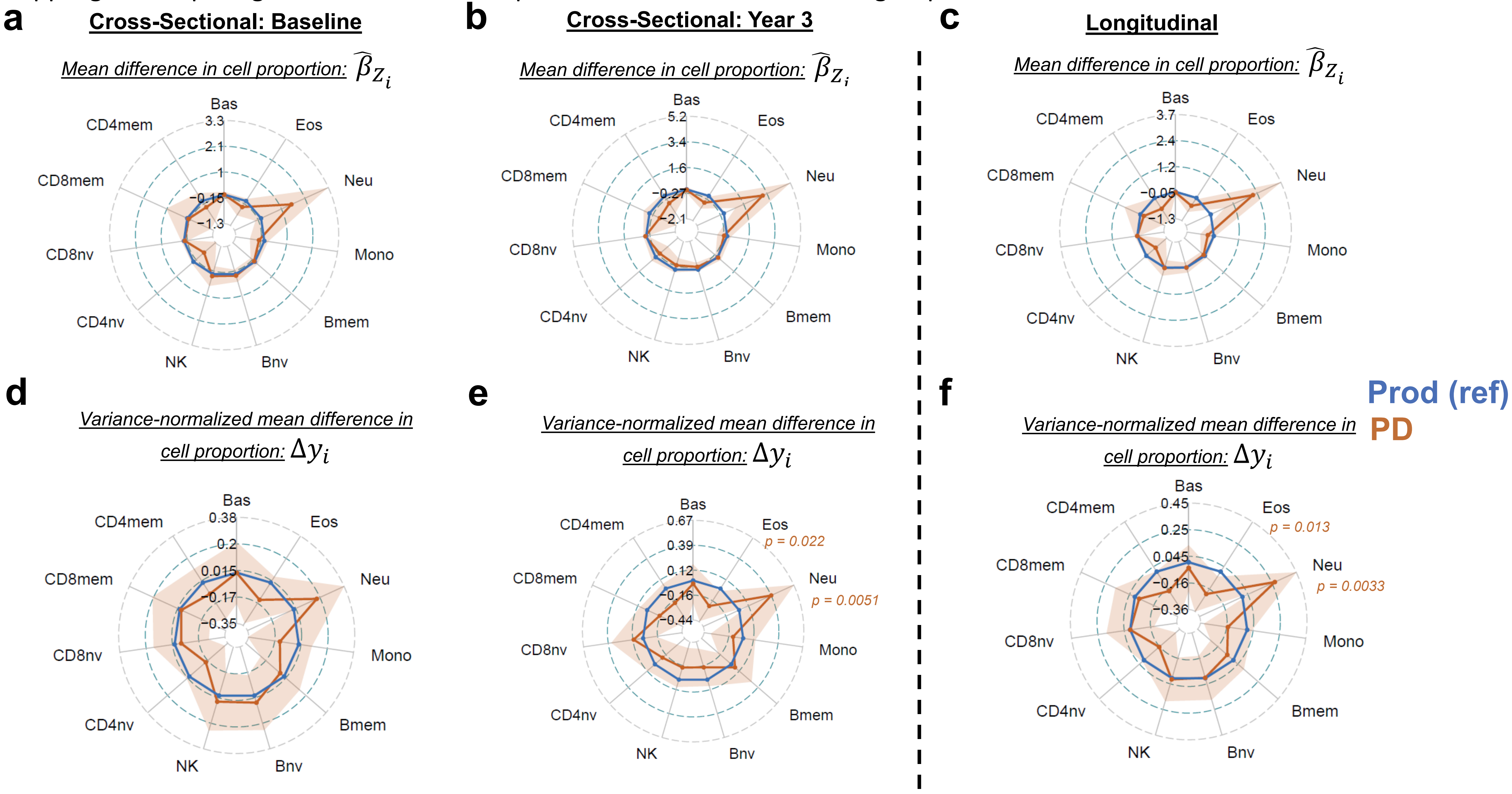

Supp Fig 4: DNAm derived immune cell proportions correlated with additional PD scores at baseline.

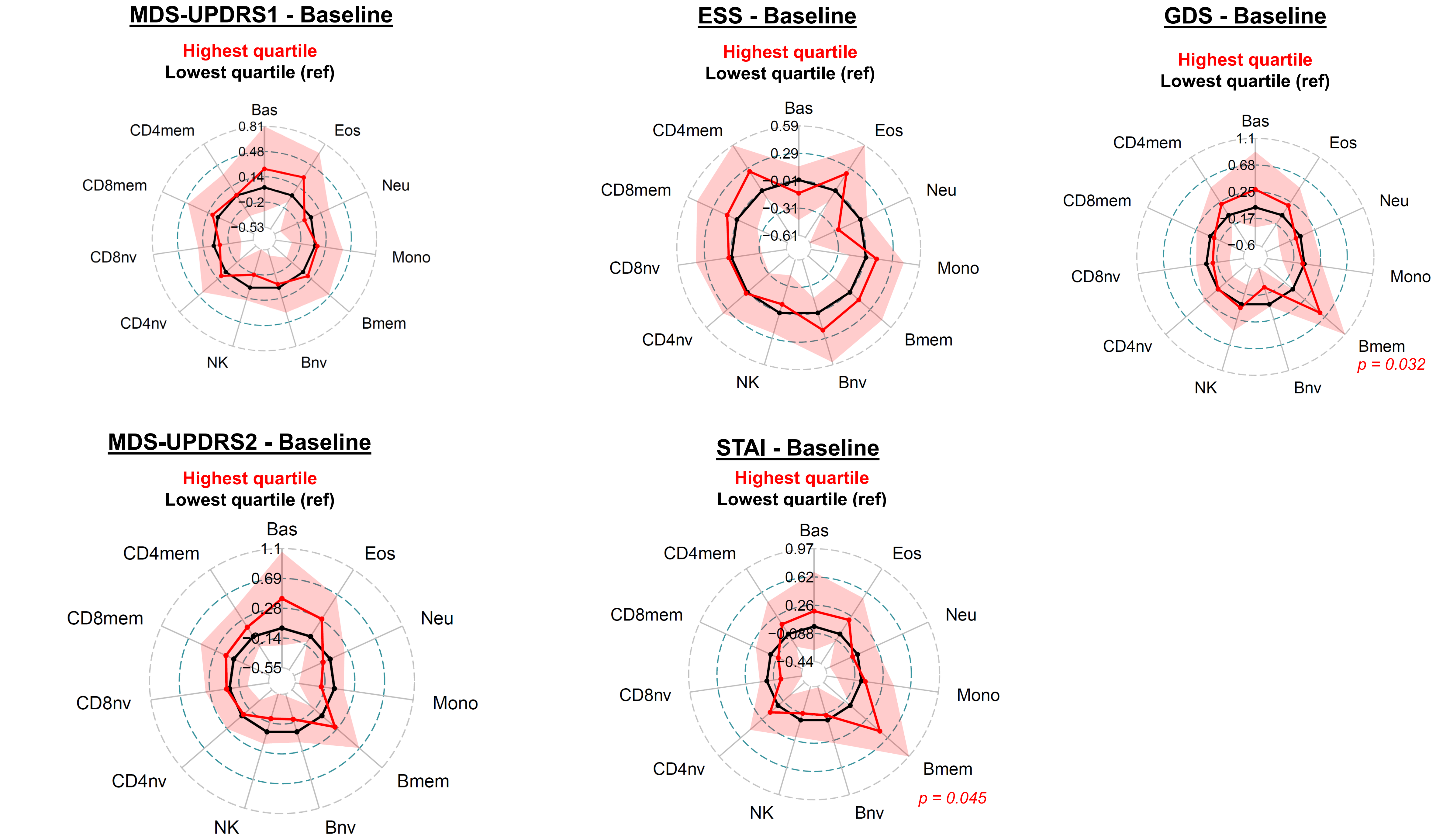

Supp Fig 5: DNAm derived immune cell proportions do not correlate with DaT scan activity at baseline.

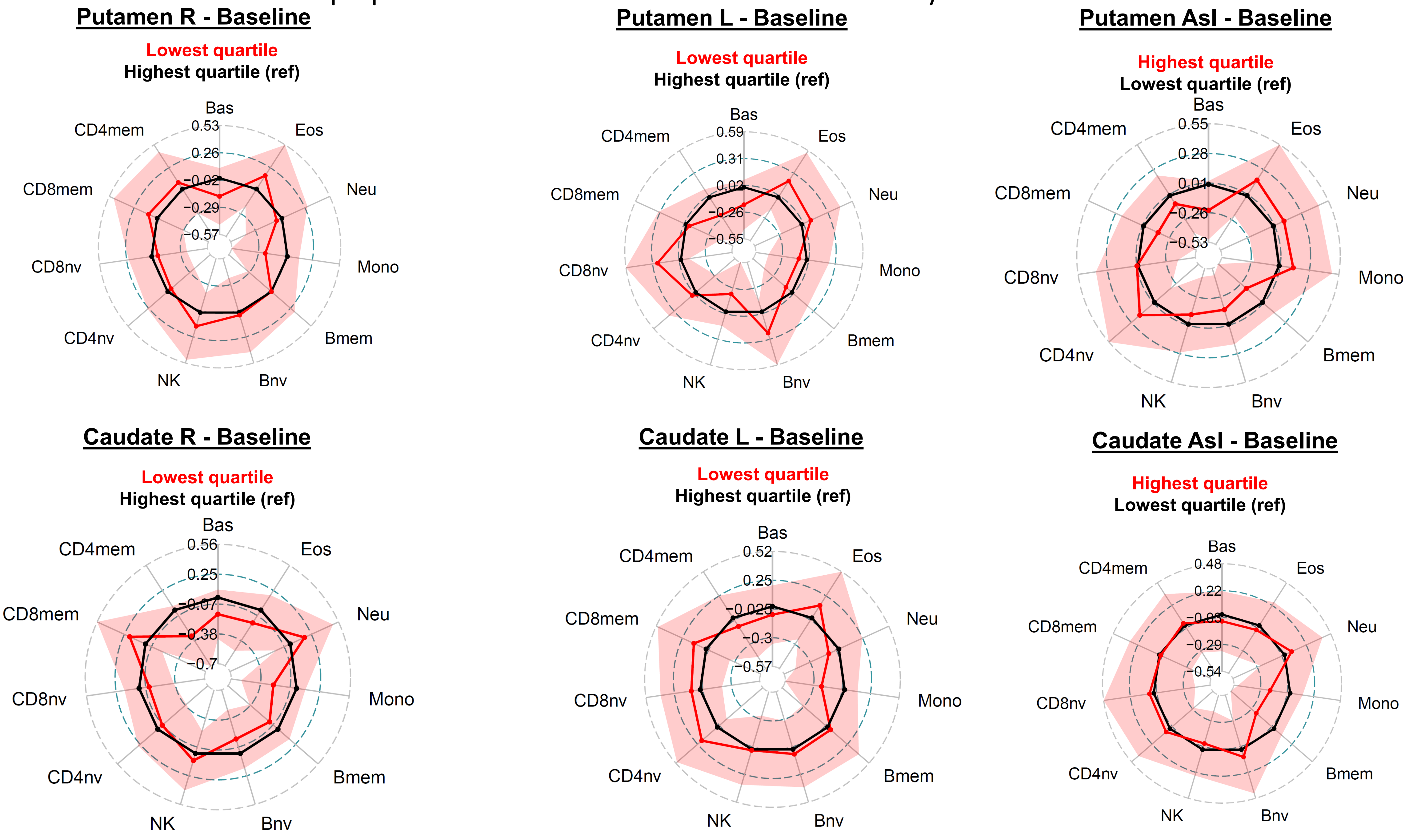

Supp Fig 6: DNAm levels at selected CpGs discovered in the EWAS analyses (part 1). . Boxplot bar represents the median value, boxplot hinge represents 25th and 75th quartiles and the whiskers represent 1.5 times the inter quartile range.

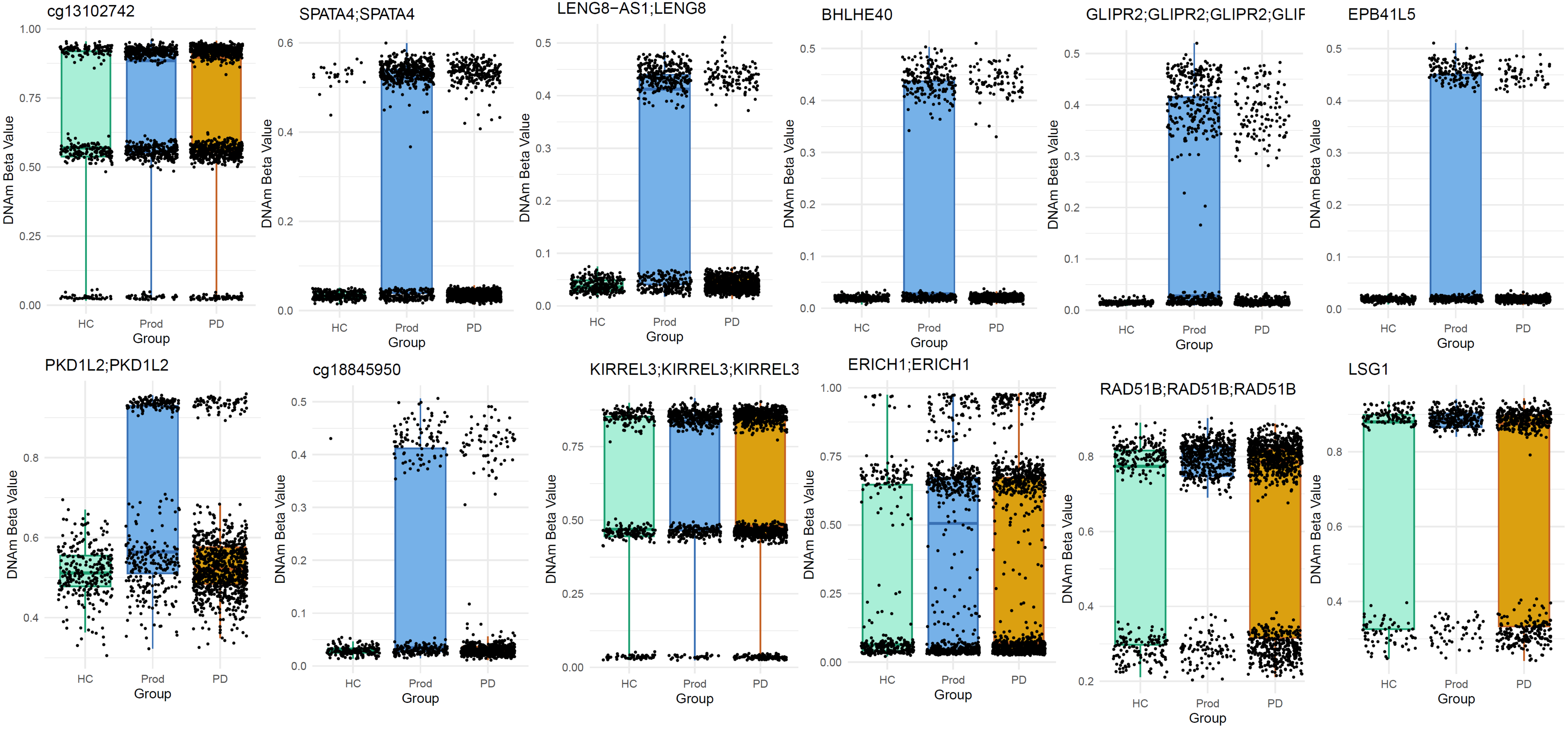

Supp Fig 7: DNAm levels at selected CpGs discovered in the EWAS analyses (part 2). . Boxplot bar represents the median value, boxplot hinge represents 25th and 75th quartiles and the whiskers represent 1.5 times the inter quartile range.

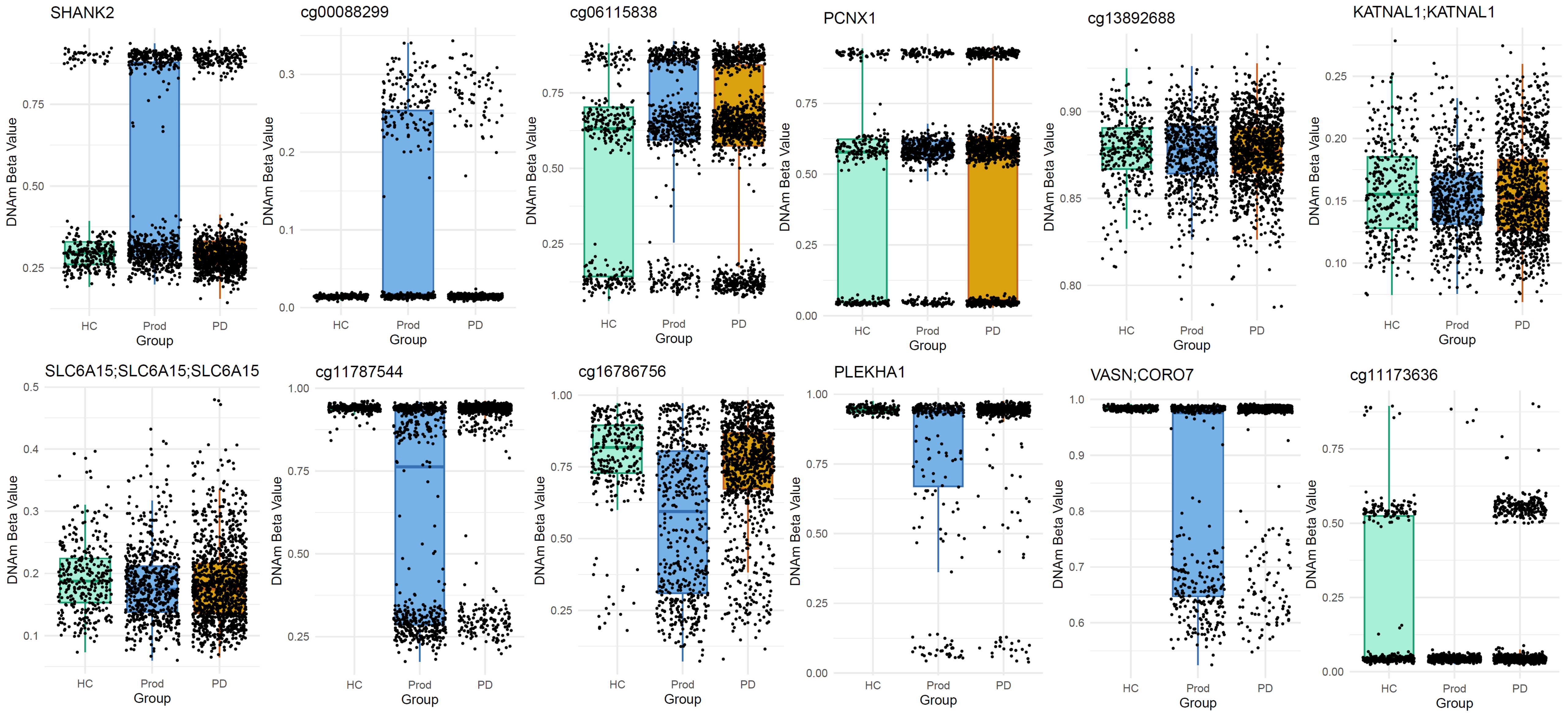

Supp Fig 8: DNAm levels at selected CpGs discovered in the EWAS analyses (part 3). . Boxplot bar represents the median value, boxplot hinge represents 25th and 75th quartiles and the whiskers represent 1.5 times the inter quartile range.

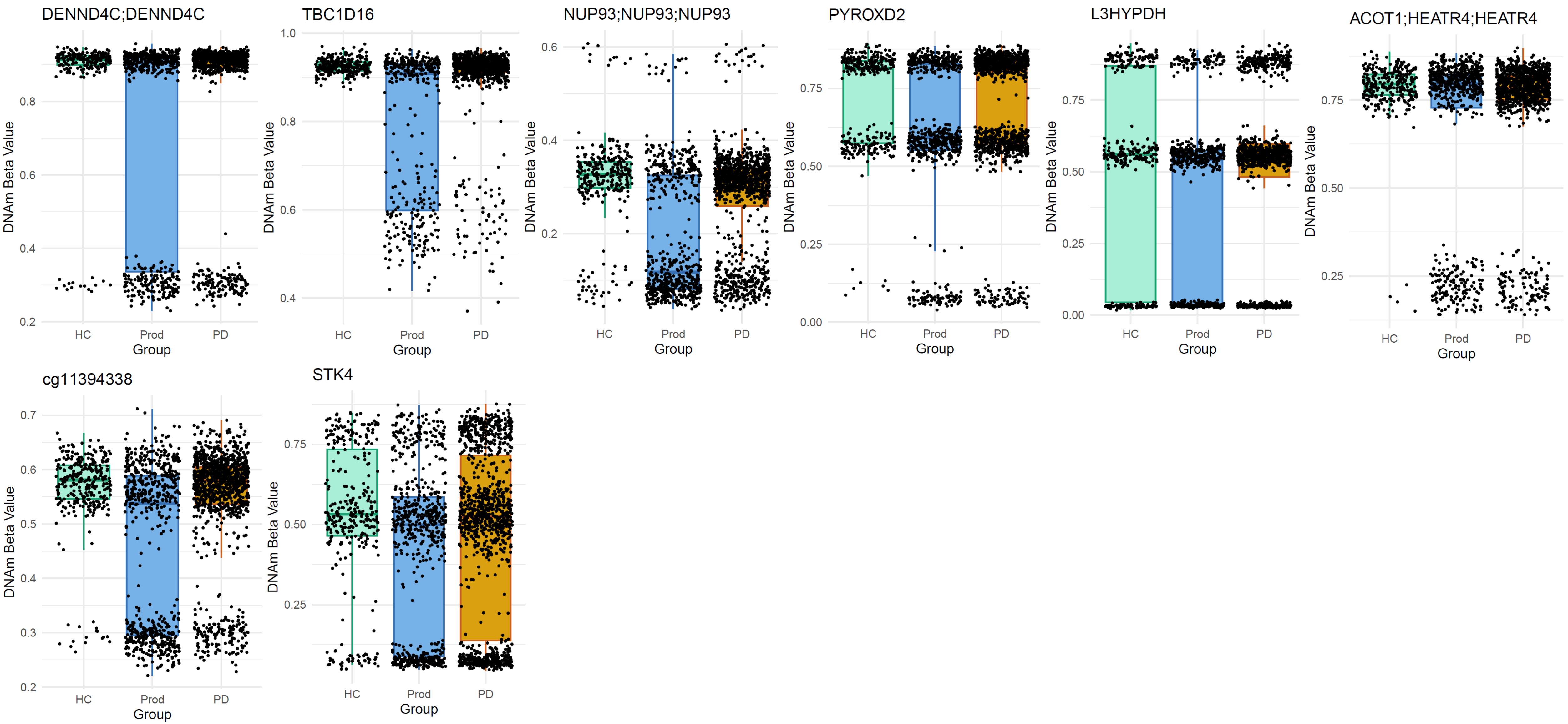

Supp Fig 9: EWAS analysis results between PD and Prodromal groups.

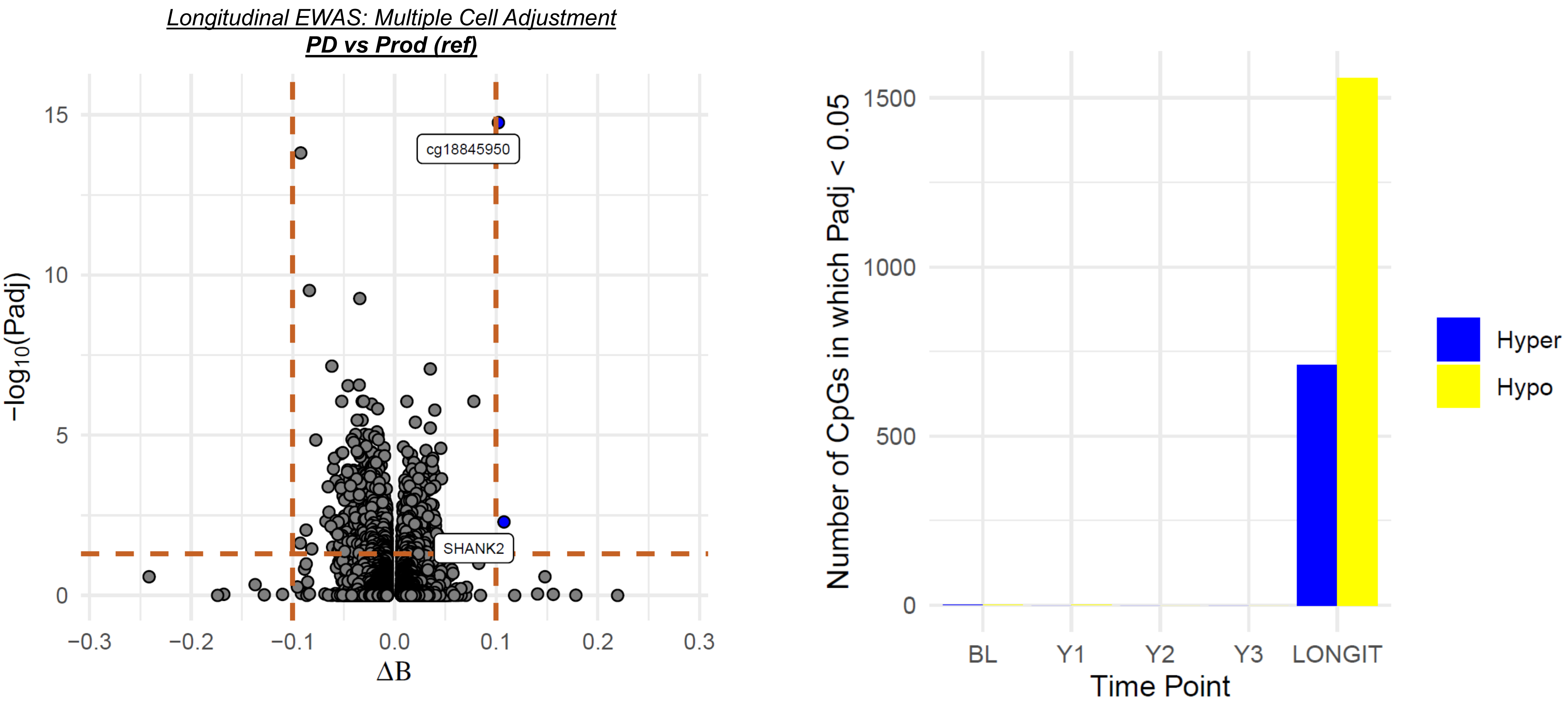

Supp Fig 10: Changes to immune cell composition observed with GBA mutational status.

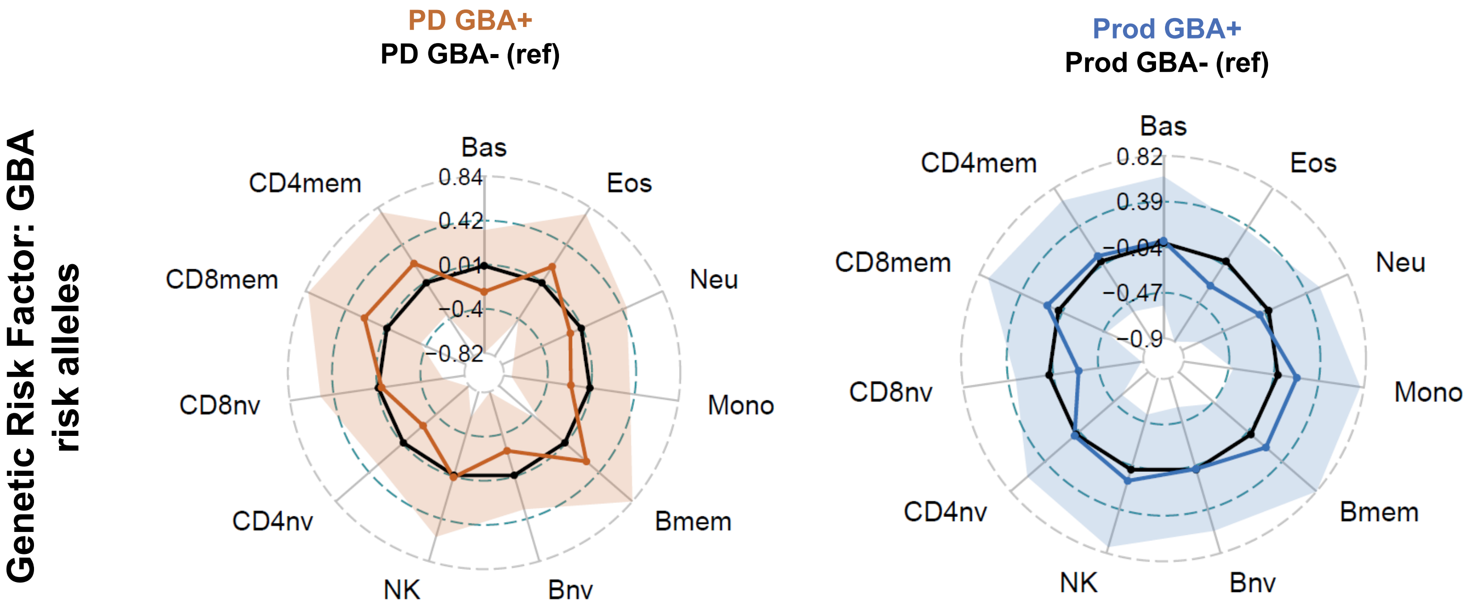

Supp Fig 11: Time to event curve for Prodromal patients who converted to PD during PPMI follow-up

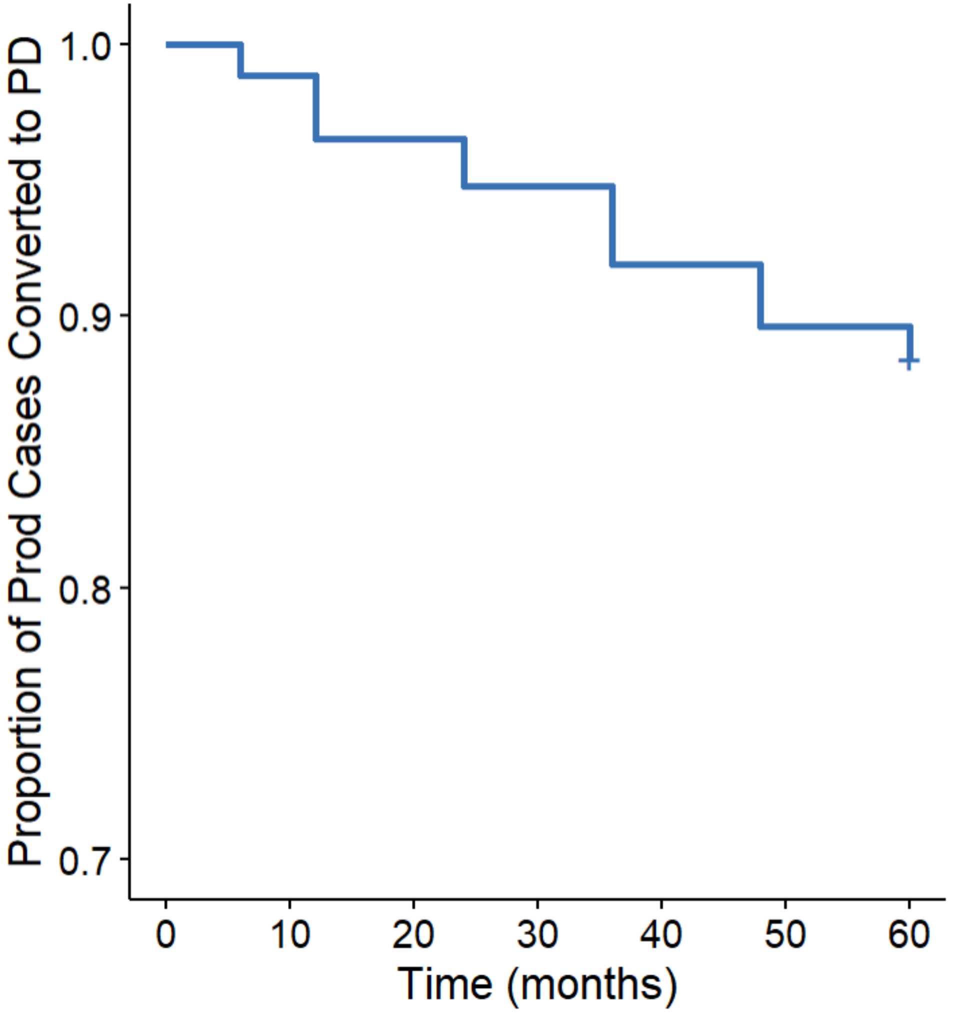

Supplement: Supplementary file 1 [file 41531_2023_626_MOESM1_ESM.pdf]
