# Supplementary material for: Immunological shifts during early-stage Parkinson’s disease identified with DNA methylation data on longitudinally collected blood samples
Source: NPJ Parkinsons Dis. 2024 Jan 11;10:21. doi: 10.1038/s41531-023-00626-6 (PMC10784484; doi:10.1038/s41531-023-00626-6)
Supplement: Supplementary file 2 — Reporting Summary [file 41531_2023_626_MOESM2_ESM.pdf]

nature portfolio

Corresponding author(s): Steven C Pike, Lucas A Salas

Last updated by author(s): Oct 30, 2023

Reporting Summary

Nature Portfolio wishes to improve the reproducibility of the work that we publish. This form provides structure for consistency and transparency in reporting. For further information on Nature Portfolio policies, see our Editorial Policies and the Editorial Policy Checklist.

Statistics

For all statistical analyses, confirm that the following items are present in the figure legend, table legend, main text, or Methods section.

n/a	Confirmed	
☐	☒	The exact sample size (*n*) for each experimental group/condition, given as a discrete number and unit of measurement
☐	☒	A statement on whether measurements were taken from distinct samples or whether the same sample was measured repeatedly
☐	☒	The statistical test(s) used AND whether they are one- or two-sided<br>*Only common tests should be described solely by name; describe more complex techniques in the Methods section.*
☐	☒	A description of all covariates tested
☐	☒	A description of any assumptions or corrections, such as tests of normality and adjustment for multiple comparisons
☐	☒	A full description of the statistical parameters including central tendency (e.g. means) or other basic estimates (e.g. regression coefficient) AND variation (e.g. standard deviation) or associated estimates of uncertainty (e.g. confidence intervals)
☐	☒	For null hypothesis testing, the test statistic (e.g. *F*, *t*, *r*) with confidence intervals, effect sizes, degrees of freedom and *P* value noted<br>*Give P values as exact values whenever suitable.*
☒	☐	For Bayesian analysis, information on the choice of priors and Markov chain Monte Carlo settings
☒	☐	For hierarchical and complex designs, identification of the appropriate level for tests and full reporting of outcomes
☐	☒	Estimates of effect sizes (e.g. Cohen's *d*, Pearson's *r*), indicating how they were calculated

Our web collection on statistics for biologists contains articles on many of the points above.

Software and code

Policy information about availability of computer code

Data collection	Data used in the preparation of this article were obtained from the Parkinson's Progression Markers Initiative (PPMI) database (www.ppmi-info.org/access-data-specimens/download-data), RRID:SCR_006431. For up-to-date information on the study, visit www.ppmi-info.org
Data analysis	All R scripts used to preprocess and analyze the data are available on our laboratory's GitHub page (https://github.com/SalasLab/PPMI_DNAm_Analysis) and on Zenodo (10.5281/zenodo.10054443).

For manuscripts utilizing custom algorithms or software that are central to the research but not yet described in published literature, software must be made available to editors and reviewers. We strongly encourage code deposition in a community repository (e.g. GitHub). See the Nature Portfolio guidelines for submitting code & software for further information.

Data

Policy information about availability of data

All manuscripts must include a data availability statement. This statement should provide the following information, where applicable:

- Accession codes, unique identifiers, or web links for publicly available datasets
- A description of any restrictions on data availability
- For clinical datasets or third party data, please ensure that the statement adheres to our policy

Data used in the preparation of this article were obtained from the Parkinson's Progression Markers Initiative (PPMI) database (www.ppmi-info.org/access-data-specimens/download-data), RRID:SCR_006431. For up-to-date information on the study, visit www.ppmi-info.org

Research involving human participants, their data, or biological material

Policy information about studies with human participants or human data. See also policy information about sex, gender (identity/presentation), and sexual orientation and race, ethnicity and racism.

Reporting on sex and gender	Sex was controlled for as a confounder in various analyses as it is a known contributor to immunological and epigenetic variance. For epigenome wide association studies, the X and Y chromosomes were not considered in the analysis.
Reporting on race, ethnicity, or other socially relevant groupings	These variables were reported but not utilized in analysis of the data.
Population characteristics	Age, Sex, diagnoses, LRRK2 and GBA genetic status, treatment status, DaT imaging results
Recruitment	The recruitment methods for the PPMI study can be found here: https://www.ppmi-info.org/study-design/research-documents-and-sops
Ethics oversight	This study was conducted in accordance with the Declaration of Helsinki and the Good Clinical Practice (GCP) guidelines after approval of the local ethics committees of the participating sites.

Note that full information on the approval of the study protocol must also be provided in the manuscript.

Field-specific reporting

Please select the one below that is the best fit for your research. If you are not sure, read the appropriate sections before making your selection.

☒ Life sciences ☐ Behavioural & social sciences ☐ Ecological, evolutionary & environmental sciences

For a reference copy of the document with all sections, see nature.com/documents/nr-reporting-summary-flat.pdf

Life sciences study design

All studies must disclose on these points even when the disclosure is negative.

Sample size	Sample size was based on availability of DNA methylation microarray data associated with the PPMI study.
Data exclusions	Poor quality DNA methylation microarray data were excluded from the analysis due to technical quality control metrics used on the Infinium DNA methylation microarrays. More information is found in Supplementary Table 1 of the manuscript.
Replication	The results we provide have been replicated within the literature using independent methods such as flow cytometry and single-cell transcriptomics. This dataset is unique in that no other similar longitudinal dataset exists on Prodromal PD patients.
Randomization	In this observational cohort study, covariates such as age and sex were included in multivariate regression models to control for these effects.
Blinding	Blinding was not relevant to this study.

Reporting for specific materials, systems and methods

We require information from authors about some types of materials, experimental systems and methods used in many studies. Here, indicate whether each material, system or method listed is relevant to your study. If you are not sure if a list item applies to your research, read the appropriate section before selecting a response.

Materials & experimental systems

n/a	Involved in the study	
☒	☐	Antibodies
☒	☐	Eukaryotic cell lines
☒	☐	Palaeontology and archaeology
☒	☐	Animals and other organisms
☐	☒	Clinical data
☒	☐	Dual use research of concern
☒	☐	Plants

Methods

n/a	Involved in the study	
☒	☐	ChIP-seq
☒	☐	Flow cytometry
☒	☐	MRI-based neuroimaging

Clinical data

Policy information about clinical studies

All manuscripts should comply with the ICMJE guidelines for publication of clinical research and a completed CONSORT checklist must be included with all submissions.

Clinical trial registration: NCT01141023

Study protocol: https://www.ppmi-info.org/study-design/research-documents-and-sops

Data collection: June 2010-June 2020 at various centers in Australia, Austria, France, Germany, Greece, Israel, Italy, Norway, Spain, United Kingdom and the United States. A full detail of each study center can be found in the clinical trial description.

Outcomes: Derived from the clinical trail description: "Mean Rates of Change [Time Frame: Baseline to 156 months] The mean rates of change and the variability around the mean of clinical, imaging and biomic outcomes in early PD patients, and where appropriate the comparison of these rates between PD patient subsets and between various subsets (including, but not limited to: PD vs. healthy subjects, PD vs. SWEDD, PD vs. prodromal, PD with and without LRRK2, GBA or SNCA mutation, unaffected LRRK2, GBA or SNCA mutation carriers vs. healthy controls) at study intervals ranging from 3 months to 36 months. Specific examples of outcomes include MDS-UPDRS, dopamine transporter imaging striatal uptake, vesicular monoamine transporter type-2 uptake, and serum and CSF alpha-synuclein. PD patient subsets may be defined by baseline assessments, genetic mutations, progression milestones and/or rate of clinical, imaging, or biomic change."

Plants

Seed stocks: *Report on the source of all seed stocks or other plant material used. If applicable, state the seed stock centre and catalogue number. If plant specimens were collected from the field, describe the collection location, date and sampling procedures.*

Novel plant genotypes: *Describe the methods by which all novel plant genotypes were produced. This includes those generated by transgenic approaches, gene editing, chemical/radiation-based mutagenesis and hybridization. For transgenic lines, describe the transformation method, the number of independent lines analyzed and the generation upon which experiments were performed. For gene-edited lines, describe the editor used, the endogenous sequence targeted for editing, the targeting guide RNA sequence (if applicable) and how the editor was applied.*

Authentication: *Describe any authentication procedures for each seed stock used or novel genotype generated. Describe any experiments used to assess the effect of a mutation and, where applicable, how potential secondary effects (e.g. second site T-DNA insertions, mosiacism, off-target gene editing) were examined.*
